# Gradients of Nigrostriatal Iron Deposition in Healthy Aging and Synucleinopathies

**DOI:** 10.1111/cns.70359

**Published:** 2025-03-25

**Authors:** Jiaqi Wen, Tao Guo, Xiaojie Duanmu, Chenqing Wu, Haoting Wu, Cheng Zhou, Qianshi Zheng, Weijin Yuan, Jianmei Qin, Zihao Zhu, Jingjing Wu, Jingwen Chen, Jingjing Xu, Yaping Yan, Jun Tian, Baorong Zhang, Hongjian He, Minming Zhang, Xiaojun Guan, Xiaojun Xu

**Affiliations:** ^1^ Department of Radiology, the Second Affiliated Hospital Zhejiang University School of Medicine Hangzhou China; ^2^ Joint Laboratory of Clinical Radiology, the Second Affiliated Hospital Zhejiang University School of Medicine Hangzhou China; ^3^ Department of Neurology, the Second Affiliated Hospital Zhejiang University School of Medicine Hangzhou China; ^4^ Center for Brain Imaging Science and Technology, College of Biomedical Engineering and Instrument Science Zhejiang University Hangzhou China

**Keywords:** gradient, iron, multiple system atrophy, Parkinson's disease, quantitative susceptibility mapping

## Abstract

**Aims:**

To investigate the gradients of nigrostriatal iron deposition in aging, Parkinson's disease (PD), and multiple system atrophy (MSA).

**Methods:**

This study included 100 young healthy controls, 171 old healthy controls (OHC), 231 PD, and 24 MSA patients. The brain iron content was quantified by quantitative susceptibility mapping. A spatial function method was employed to map the iron gradient along the principal axis of the subcortical structure. General linear models were used to compare differences in iron gradients between groups. Partial correlation was used to analyze the relationship between iron content and symptoms of synucleinopathies.

**Results:**

Nigrostriatal iron deposition in all gradient directions was observed during aging (*p* < 0.05). Compared to OHC, iron deposition was significant in nearly all substantia nigra (SN) segments in both PD and MSA (*p* < 0.05). MSA showed significant iron deposition in the posterolateral putamen compared to PD (*p* < 0.05). Iron deposition in the SN in PD and putamen in MSA correlated with disease severity.

**Conclusion:**

Iron deposition in all gradient directions occurred in the nigrostriatal system during healthy aging, and this was more evident in the SN in both PD and MSA, with MSA displaying additional iron deposition in the posterolateral putamen.

## Graphical abstract

Nigrostriatal iron gradients are similar in HC, PD, and MSA, with variation in extent. Iron deposition in the SN emerged as a consistent feature in synucleinopathies. MSA has a higher iron content in the posterolateral putamen compared to PD.

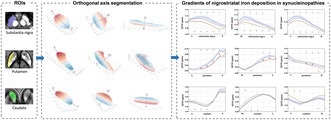

## 1 Introduction

Parkinson's disease (PD) and multiple system atrophy (MSA) are classified as synucleinopathies, in which age is a significant risk factor [1, 2]. The core pathology of PD and MSA involves the aggregation of misfolded α‐synuclein in neurons and oligodendrocytes, respectively [3, 4], with the nigrostriatal system being a primary target [5]. Clinically, PD and MSA patients share similar parkinsonism motor symptoms including bradykinesia, rigidity, and tremor. Additionally, MSA patients present more significant autonomic dysfunction, rapid disease progression, and shorter survival time [6]. It is evident that PD and MSA overlap in pathological and clinical features. Until now, the distinct patterns of nigrostriatal neurodegeneration that differentiate these two conditions, as well as their connection to clinical symptoms, are yet to be fully understood.

Non‐heme iron is essential for maintaining normal cellular functions and participating in key physiological processes such as neurotransmitter synthesis, synaptic plasticity, and myelination [2]. Iron homeostasis is critical for normal brain function, yet it is disrupted in healthy aging and neurodegenerative diseases, leading to non‐heme iron accumulation outside storage complexes [2], which in turn triggers inflammation and oxidative stress that damage cellular components [7]. Post‐mortem and in vivo studies have demonstrated selective brain iron deposition during healthy aging, particularly in the nigrostriatal system [7, 8]. Pathophysiologically, excessive iron deposition in the substantia nigra (SN) could facilitate α‐synuclein fibril formation and induce ferroptosis of neurons [9]. Increased iron concentration was observed in the SN in PD and in the putamen in MSA, respectively [10, 11, 12]. Nigrostriatal iron deposition can be considered a common pathophysiological feature in healthy aging and synucleinopathies (PD and MSA). Notably, the inferior SN of PD patients and the posterolateral putamen of MSA patients showed more pronounced iron deposition relative to other regions of the same nucleus [13, 14], implicating inhomogeneity of iron deposition within the nigrostriatal system [15]. The symptomatic manifestations of synucleinopathies may be related to the selective deposition of iron within the nuclei. Therefore, it is necessary to explore the spatial gradients of iron deposition in this system in these synucleinopathies.

Quantitative susceptibility mapping (QSM) is an advanced magnetic resonance imaging (MRI) technique that accurately quantifies the spatial distribution of tissue magnetic susceptibility, becoming the gold standard for in vivo quantification of brain iron [16]. QSM has been widely applied to explore brain iron content changes in healthy aging and age‐related neurodegenerative diseases [17, 18]. Previous studies have employed various methods to segment subcortical gray matter nuclei to explore the uneven iron deposition within these structures. It has been observed that iron deposition in the inferior and posterior regions of the SN is more pronounced compared to other areas [13, 19]. A meta‐analysis revealed increased QSM values at both the SN pars compacta and SN pars reticulata levels in patients with PD [20]. These prior studies, while investigating iron deposition in SN subregions, utilized a single‐dimensional spatial segmentation approach that inadequately revealed the uneven distribution of iron within the nuclei and overlooked the specific morphological features of the nuclei. The region of interest (ROI) principal axis‐based spatial segmentation method, employing a singular value decomposition (SVD) algorithm, redefines the three‐dimensional coordinates of the nuclei based on the morphological structure and enables the performance of equidistant segmentation. A previous study has demonstrated the presence of microstructural gradients in the striatum and their relevance to aging and PD, revealing posterior putaminal degeneration linked to motor dysfunction [21]. However, this study primarily focused on characterizing structural gradients using multiparametric quantitative MRI without quantifying iron deposition gradients. In conjunction with QSM technology, this method enables comprehensive quantification of iron deposition gradients with specificity to the nuclei [21].

In the present study, we integrate QSM with an ROI axis‐based spatial segmentation approach to investigate the iron gradients in the nigrostriatal system during healthy aging and in different types of age‐related synucleinopathies and further explore their impacts on clinical symptoms. We hypothesize that although both PD and MSA involve the nigrostriatal system, the gradients of iron deposition in these two synucleinopathies may differ, which may potentially correlate with specific clinical symptoms.

## 2 Materials and Methods

### 2.1 Participants

All NC, PD, and MSA patients signed informed consent forms in accordance with the approval of the Medical Ethics Committee of the Second Affiliated Hospital of Zhejiang University School of Medicine (ethical approval numbers: 2012–26 and 2017–008). An experienced neurologist (B.Z.) diagnosed PD according to the UK Parkinson's Disease Society Brain Bank criteria [22] and MDS clinical diagnostic criteria for Parkinson's disease [23], and diagnosed MSA according to the Movement Disorder Society Criteria for the Diagnosis of Multiple System Atrophy [14].

This study initially recruited 100 young healthy controls (YHC), 226 old healthy controls (OHC), 347 PD patients, and 26 MSA patients. Exclusion criteria included (1) other neurological or psychiatric disorders, (2) history of traumatic brain injury, (3) claustrophobia, (4) presence of a cardiac pacemaker or any other internal metal implants incompatible with MRI, and (5) significant brain abnormalities detected during MRI examinations, such as severe brain atrophy, hydrocephalus, brain tumors, and cerebral hemorrhage. Additionally, images with noticeable head artifacts were excluded based on visual inspection. Sex and age were matched between groups. Ultimately, a total of 100 YHC, 171 OHC, 231 PD, and 24 MSA patients (14 with MSA‐C and 10 with MSA‐P) were included in the following analyses [14].

Among the 231 PD patients included in this study, 87 were drug‐naïve, while the remaining 144 patients were receiving optimized antiparkinsonian medications based on clinical recommendations. Their medication regimens included either a single drug or a combination of levodopa, dopamine agonists (pramipexole dihydrochloride), amantadine, catechol‐O‐methyl transferase inhibitors, monoamine oxidase B inhibitors (selegiline), and trihexyphenidyl. For PD patients receiving antiparkinsonian treatment, clinical assessments, and MRI scans were conducted in the morning following the withdrawal of all antiparkinsonian medications overnight (for at least 12 h, referred to as the ‘drug‐off status’). Additionally, we obtained on‐state UPDRS part III scores from a subset of 104 PD patients. The on‐state is defined as 1 h after the levodopa challenge test (administration of 200 mg levodopa and 50 mg benserazide). We collected essential demographic data, including age, sex, and education levels of all participants, and also documented the disease duration in PD and MSA patients. Additionally, the Hoehn‐Yahr stage of PD patients and the subtype of MSA patients were recorded. OHC, PD, and MSA patients underwent evaluations using the Mini‐Mental State Examination (MMSE) and the Scales for Outcomes in Parkinson's Disease—Autonomic (SCOPA‐AUT). Additionally, PD and MSA patients were assessed symptoms using the Unified Parkinson's Disease Rating Scale (UPDRS) and the Unified Multiple System Atrophy Rating Scale (UMSARS), respectively.

### 2.2 MRI Data Acquisition

All participants underwent scanning on a 3.0‐Tesla MRI scanner (GE Discovery 750) with an 8‐channel head coil. To ensure stability during MRI scanning, the head was secured using foam pads, and earplugs were provided to minimize noise interference. Enhanced susceptibility‐weighted angiography (ESWAN) was acquired to generate QSM. The ESWAN images were acquired using a gradient recalled echo sequence. Sequence parameters are detailed in Appendix S1.

### 2.3 QSM Data Processing and ROIs Segmentation

**FIGURE 1:**
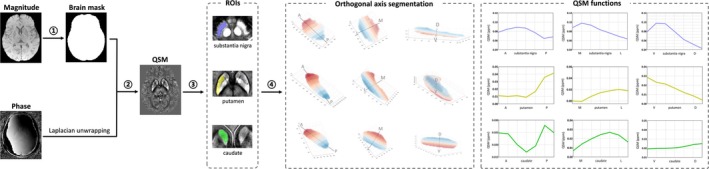
Flow chart of QSM image reconstruction, ROIs segmentation, and QSM functions generation. The ESWAN images, including magnitude and phase images, were acquired from gradient recalled echo sequence. The magnitude images were used to generate a brain mask (①). The QSM image was reconstructed by using unwrapped phase images and the brain mask (②). The ROIs (substantia nigra, putamen, and caudate nucleus) were segmented on the QSM image utilizing DeepQSMSeg (③). The ROI axis‐based spatial segmentation approach (https://github.com/MezerLab/mrGrad) was used to segment ROIs equidistant along three orthogonal axes, and QSM functions were finally generated (④). A, Anterior; D, Dorsal; ESWAN, Enhanced susceptibility‐weighted angiography; L, Lateral; M, Medial; *P*, Posterior; QSM, Quantitative susceptibility mapping; ROIs, Regions of interest; V, Ventral.

Susceptibility Tensor Imaging Suite V3.0 software package (https://people.eecs.berkeley.edu/~chunlei.liu/software.html) was used to calculate the susceptibility maps from the phase images [16, 24, 25, 26]. Detailed processing procedures are provided in Figure 1 and Appendix S2.

A Deep Learning‐based Sub‐cortical Nucleus Segmentation Tool (DeepQSMSeg) was utilized to automatically segment ROIs including SN, putamen, and caudate nucleus in the native space [27]. An experienced radiologist (X.G.) visually inspected all automatically segmented data and manually corrected it to ensure segmentation accuracy.

### 2.4 QSM Gradients Mapping of Subcortical Nuclei

We employed an automated procedure (https://github.com/MezerLab/mrGrad) [21] to generate QSM functions along the principal axes of a subcortical structure at the individual subject level. The input data were all participants' native QSM images, along with the ROI masks segmented by DeepQSMSeg and manually corrected. Without the need for image rotation, this method transitioned from image axes to ROI‐based axes and then extracted QSM values as a function of their spatial positions. The bilateral mean QSM gradients of SN, putamen, and caudate nucleus were reported (Figure 1).

#### 2.4.1 Orthogonal Axis Calculation

With a given ROI mask, the algorithm calculates the SVD of the voxel 3D image coordinates within the ROI to determine the principal three orthogonal axes of the structure [21]. In the present study, three orthogonal axes of the SN, putamen, and caudate nucleus were identified as the anterior–posterior (AP), ventral–dorsal (VD), and medial–lateral (ML) axes. The SVD algorithm solution was described in detail in Appendix S3.

#### 2.4.2 Orthogonal Axis Segmentation

We partitioned the ROI with uniform spacing for each of the three principal orthogonal axes. To achieve this, we designated the data edges as the two hyperplanes determined by the two outermost data points relative to the axis, with the axis as normal to the plane. Subsequently, the data were divided by *n–*1 parallel hyperplanes evenly distributed between the two data edges. Voxels were then categorized into n segments. In our study, we opted for *n* = 7 for segmentation, based on previous studies that used this method to segment subcortical nuclei [21] (Figure 1).

#### 2.4.3 QSM Functions Along the Orthogonal Axes of the ROI

Given a QSM map, we calculated a median value for each segment along every axis. This results in three ROI‐based functions representing spatial positions along the three principal orthogonal axes of the designated ROI. These spatial functions were then averaged across subjects for each axis to establish group‐averaged functions of spatial variations within the ROI.

### 2.5 Statistical Analyses

A normality test was conducted for each group of data. The Shapiro–Wilk test was adopted when the sample size was equal to or less than 50, and the Kolmogorov–Smirnov test was adopted when the sample size was more than 50.

The chi‐square test was used for pairwise comparison of sex differences between groups. When comparing age and education between YHC and OHC, and disease course between PD and MSA, the two independent samples T‐test was used for data with normal distribution, and the Mann–Whitney U test was used for data with non‐normal distribution. The general linear model was used to compare age, education, MMSE, and SCOPA‐AUT scores between OHC, PD, and MSA, with additional correction for sex and age when comparing MMSE and SCOPA‐AUT scores. Then, post hoc with LSD correction was used to test the difference between any two groups.

A general linear model was used to compare the difference in nuclear iron gradients between YHC and OHC by controlling for sex to explore brain iron deposition during healthy aging [28, 29, 30, 31]. A general linear model was used to explore intergroup differences in the iron gradients among OHC, PD, and MSA by controlling for sex and age to investigate brain iron deposition in age‐related synucleinopathies. P values were adjusted for the false discovery rate (FDR) to control for false positive rates due to multiple comparisons.

Partial correlation analysis was used to analyze the relationship between iron content in ROI segments with significantly elevated iron deposition compared to OHC and clinical symptoms in the disease group, controlling for sex and age.

## 3 Results

### 3.1 Demographic and Clinical Characteristics of the Study Population

Demographic and clinical information is detailed in Table 1. There was no significant difference in age among the OHC, PD, and MSA groups (*F* = 0.505, *p*_GLM_ = 0.604). Sex distribution did not significantly differ among the four groups (OHC vs. YHC, *p* = 0.460; OHC vs. PD, *p* = 0.110; OHC vs. MSA, *p* = 0.984; PD vs. MSA, *p* = 0.464). While there was no significant distinction in education level between PD and MSA (*p* = 0.533), notable differences in education level were observed among the other groups (OHC vs. YHC, *p* < 0.001; OHC vs. PD, p < 0.001; OHC vs. MSA, *p* = 0.003).

**TABLE 1:** Overview of demographic and clinical data of participants.

	YHC	OHC	PD	MSA	*p*_1_	*p*_2_	*p*_3_	*p*_4_	*F*/*p*_*GLM*_
**Demographic data**									
Number (n)	100	171	231	24	—	—	—	—	—
Age (years)	24.00 [2.00]	60.40 [10.27]	59.74 ± 8.89	61.31 ± 6.47	**< 0.001**	0.547	0.539	0.364	0.505/0.604
Sex (male/female)	41/59	78/93	124/107	11/13	0.460	0.110	0.984	0.464	—
Education (years)	17.00 [1.00]	10.00 [4.00]	8.00 [6.00]	7.00 ± 4.22	**< 0.001**	**< 0.001**	**0.003**	0.533	16.152/**< 0.001**
Disease duration (years)	—	—	2.54 [3.87]	2.64 ± 1.75	—	—	—	0.336	—
MSA subtype (MSA‐C/MSA‐P)	—	—	—	14/10	—	—	—	—	—
**Clinical data**									
Hoehn‐Yahr stage	—	—	2.5 [0.5]	—	—	—	—	—	—
MMSE	—	29.00 [3.00]	27.00 [4.00]	25.00 ± 3.66	—	**< 0.001**	**< 0.001**	0.295	23.725/**< 0.001**
SCOPA‐AUT	—	4.00 [4.00]	8.00 [7.00]	18.96 ± 9.58	—	**< 0.001**	**< 0.001**	**< 0.001**	81.214/**< 0.001**
UPDRS part I	—	—	1.00 [2.00]	—	—	—	—	—	—
UPDRS part II	—	—	8.00 [6.00]	—	—	—	—	—	—
UPDRS part III (off)	—	—	20.00 [18.00]	—	—	—	—	—	—
UPDRS part III (on)	—	—	11.00 [12.00]	—	—	—	—	—	—
UMSARS part I	—	—	—	12.33 ± 5.49	—	—	—	—	—
UMSARS part II	—	—	—	16.67 ± 6.55	—	—	—	—	—
UMSARS part III									
Supine	—	—	—	139/86 (70)	—	—	—	—	—
Standing (instant)	—	—	—	110/73 (82)	—	—	—	—	—
Standing (1 min)	—	—	—	110/74 (82)	—	—	—	—	—
Standing (3 min)	—	—	—	110/74 (82)	—	—	—	—	—
Standing (5 min)	—	—	—	108/72 (82)	—	—	—	—	—
UMSARS part IV	—	—	—	1.00 [2.00]	—	—	—	—	—

*Note:* Data with normal distribution was expressed as mean ± SD, data with non‐normal distribution was expressed as median [IQR], and UMSARS part III data was expressed as systolic/diastolic blood pressure (heart rate). *p*‐values for statistically significant differences are shown in bold.

Abbreviations: *F*, the test statistic of GLM; GLM, general linear model; MMSE, Mini‐Mental State Examination; MSA, Multiple system atrophy; OHC, Old healthy controls; *p*_1_, differences between YHC and OHC; *p*_2_, differences between OHC and PD; *p*_3_, differences between OHC and MSA; *p*_4_, differences between PD and MSA; PD, Parkinson's disease; *p*_GLM_, *p*‐value of the general linear model for comparison between OHC, PD, and MSA; SCOPA‐AUT, Scales for Outcomes in Parkinson's disease ‐Autonomic; UMSARS, Unified Multiple System Atrophy Rating Scale; UPDRS, Unified Parkinson's Disease Rating Scale; YHC, Yong healthy controls.

The MMSE scores in PD and MSA were significantly lower than those in OHC (OHC vs. PD, *p* < 0.001; OHC vs. MSA, *p* < 0.001); no significant difference was observed between PD and MSA (*p* = 0.295). In terms of SCOPA‐AUT scores, both PD and MSA showed a significant decrease compared to OHC (OHC vs. PD, *p* < 0.001; OHC vs. MSA, *p* < 0.001), with MSA demonstrating notably lower scores compared to PD (*p* < 0.001). UMSARS part III revealed the presence of orthostatic hypotension in MSA patients.

### 3.2 Iron Deposition Gradient in the Nigrostriatal System During Healthy Aging

**FIGURE 2:**
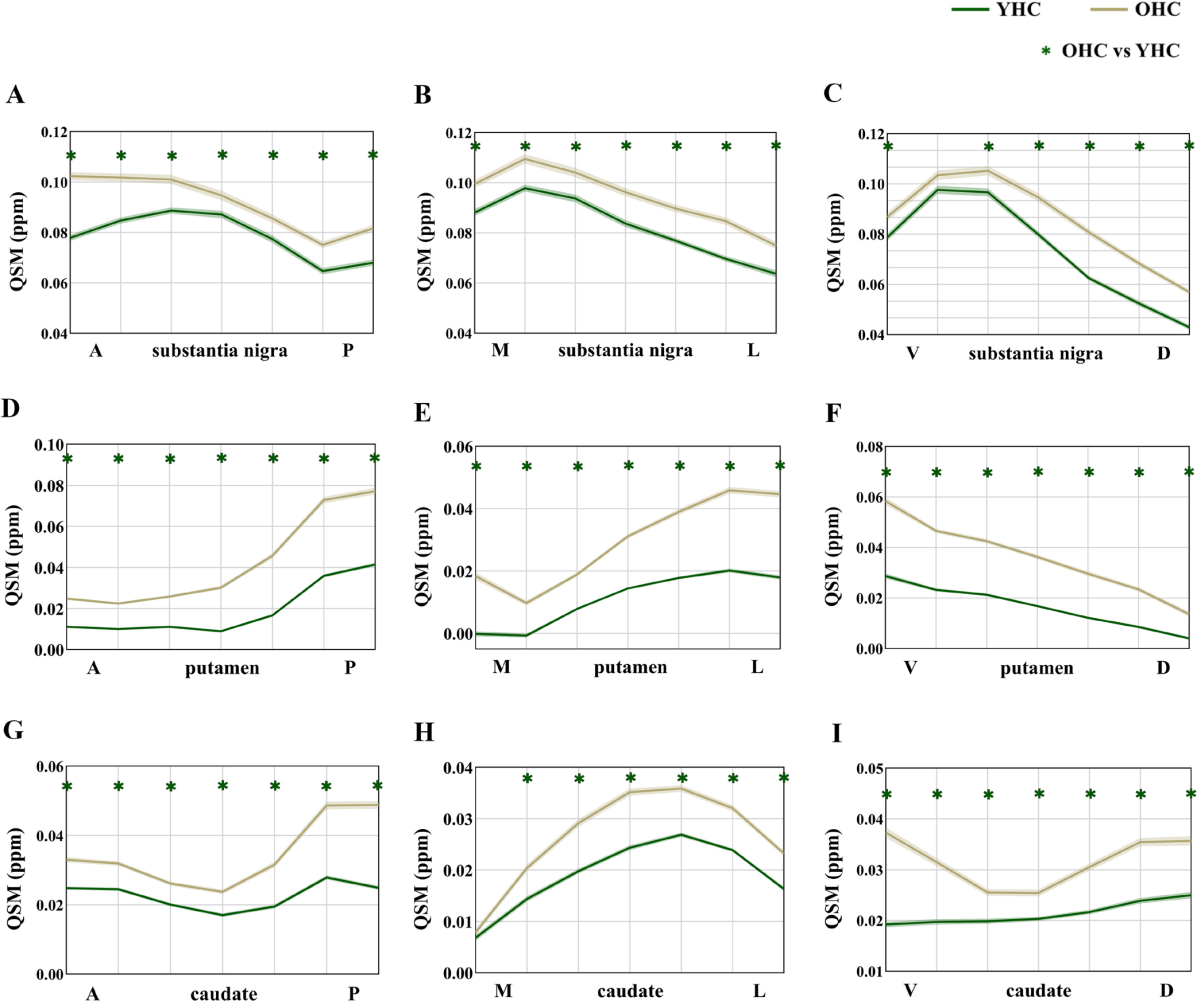
Iron deposition gradients in the nigrostriatal system during healthy aging. (A–C). Gradients of nigral iron deposition in the principal axes of YHC and OHC; (D–F). Gradients of putamen iron deposition in the principal axes of YHC and OHC; (G–I). Gradients of caudate nucleus iron deposition in the principal axes of YHC and OHC. During healthy aging, significant iron deposition was detected in all segments of the SN, caudate nucleus, and putamen, except for segment 2 of the SN‐VD axis and segment 1 of the caudate‐ML axis. Curves represent the mean. Shaded areas represent ±1 SEM. The green line represents YHC and the yellow line represents OHC. Green * represents segments with statistically significant group differences in iron content. A, Anterior; D, Dorsal; L, Lateral; M, Medial; OHC, Old healthy controls; *P*, Posterior; QSM, Quantitative susceptibility mapping; V, Ventral; YHC, Young healthy controls.

The gradient of iron deposition differed along each ROI axis of the SN and striatum. Specifically, an inverted U‐shaped pattern was observed along the SN‐ML, SN‐VD, and caudate‐ML axes (Figure 2B,C,H). The iron distribution along the SN‐AP, putamen‐ML, and caudate‐AP axes exhibited an S‐shaped or inverted S‐shaped pattern (Figure 2A,E,G). In the putamen‐AP and putamen‐VD axes, the iron distribution displayed a gradual increase and decrease, respectively (Figure 2D,F). In the caudate‐VD axis, YHC presented a gradual increase in iron distribution and OHC presented a U‐shaped iron distribution (Figure 2I).

During healthy aging, significant iron deposition was detected in all segments of the SN, caudate nucleus, and putamen (*p*_FDR_ < 0.05), except for segment 2 of the SN‐VD axis (*p*_FDR_ = 0.051) and segment 1 of the caudate‐ML axis (*p*_FDR_ = 0.175) (Figure 2, detailed statistics can be found in Appendix S4).

### 3.3 Iron Deposition Gradient in the Nigrostriatal System of the Synucleinopathies

**FIGURE 3:**
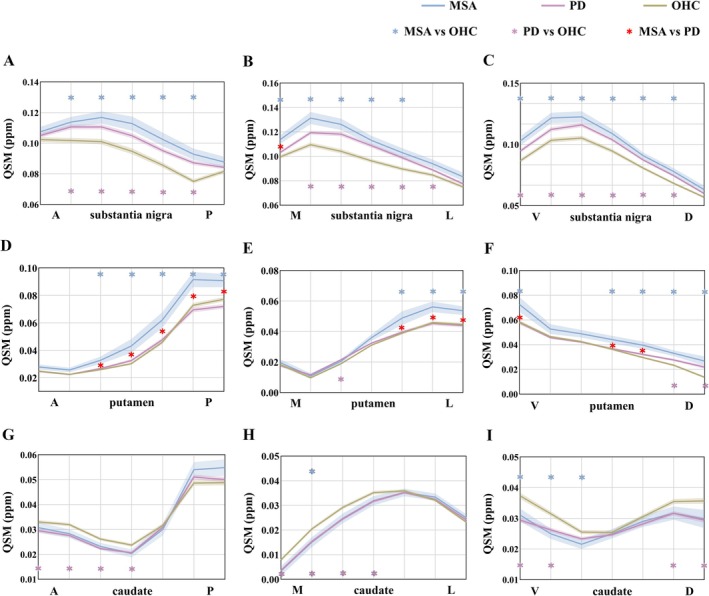
Iron deposition gradients in the nigrostriatal system in synucleinopathies. (A–C). Gradients of nigral iron deposition in three principal axes of OHC, PD, and MSA; (D–F). Gradients of putamen iron deposition in three principal axes of OHC, PD, and MSA; (G–I). Gradients of caudate nucleus iron deposition in three principal axes of OHC, PD, and MSA. Compared to OHC, iron deposition was significant in nearly all SN segments in both PD and MSA. MSA showed significant iron deposition in the posterolateral putamen compared to PD and OHC. Curves represent the mean. Shaded areas represent ±1 SEM. The blue line represents MSA patients; The purple line represents PD patients. The yellow line represents the healthy elderly. Blue * represents the segments with statistically significant differences in iron deposition between MSA patients and healthy elderly. Purple * represents the segments with statistically significant differences in iron deposition between PD patients and healthy elderly. Red * represents the segments with statistically significant differences in iron deposition between MSA and PD patients. A, Anterior; D, Dorsal; L, Lateral; M, Medial; MSA, Multiple system atrophy; OHC, Old healthy controls; *P*, Posterior; QSM, Quantitative susceptibility mapping; V, Ventral; YHC, Young healthy controls.

Iron deposition of the nigrostriatal system in PD and MSA showed a similar gradient distribution to OHC but with different extents. In PD and MSA, significant iron deposition was observed in most segments of the SN compared to OHC (*p*_FDR_ < 0.05), except for segments 1 and 7 of the SN‐AP axis, segments 1 and 7 of the SN‐ML axis, segment 7 of the SN‐VD axis in PD, as well as segments 1 and 7 of the SN‐AP axis, segments 6 and 7 of the SN‐ML axis, and segment 7 of the SN‐VD axis in MSA (*p*_FDR_ > 0.05). Moreover, iron deposition in MSA was notably higher than in PD in segment 1 of the SN‐ML axis (*p*_FDR_ = 0.037). (Figure 3A–C, detailed statistics can be found in Appendix S5).

In the putamen, compared to OHC, individuals with MSA exhibited significant iron deposition in the posterolateral putamen (segments 3–7 of the putamen‐AP axis, *p*_FDR_ = 0.012, 0.001, 0.001, 0.004, 0.025; segments 5–7 of the putamen‐ML axis, *p*_FDR_ = 0.006, 0.011, 0.032). These segments also displayed higher iron levels than those in PD (segments 3–7 of the putamen‐AP axis, *p*_FDR_ = 0.040, 0.008, 0.007, 0.001, 0.002; segments 5–7 of the putamen‐ML axis, *p*_FDR_ = 0.013, 0.009, 0.022, respectively). In PD, significant iron deposition was only observed in segment 3 of the putamen‐ML axis and segments 6 and 7 of the putamen‐VD axis compared to OHC (*p*_FDR_ < 0.05). (Figure 3D–F, detailed statistics can be found in Appendix S5).

In the caudate nucleus, iron deposition was lower in PD and MSA than in OHC. Specifically, for PD, the iron content of segments 1–4 of the caudate‐AP axis (*p*_FDR_ = 0.006, < 0.001, 0.001, 0.009), segments 1–4 of the caudate‐ML axis (*p*_FDR_ < 0.001, < 0.001, < 0.001, = 0.005), and segments 1,2,6,7 of the caudate‐VD axis (P_FDR_ < 0.001, < 0.001, = 0.007, < 0.001) was lower than OHC; for MSA, the iron content of segment 2 of the caudate‐ML axis (*p*_fdr_ = 0.020) and segments 1–3 of the caudate‐VD axis was lower than OHC (P_FDR_ = 0.030, 0.008, 0.043). The caudate nucleus showed no significant differences in iron content between PD and MSA. (Figure 3G–I, detailed statistics can be found in Appendix S5).

### 3.4 Relationship Between Nigrostriatal Iron Deposition and Symptoms of Synucleinopathies

**FIGURE 4:**
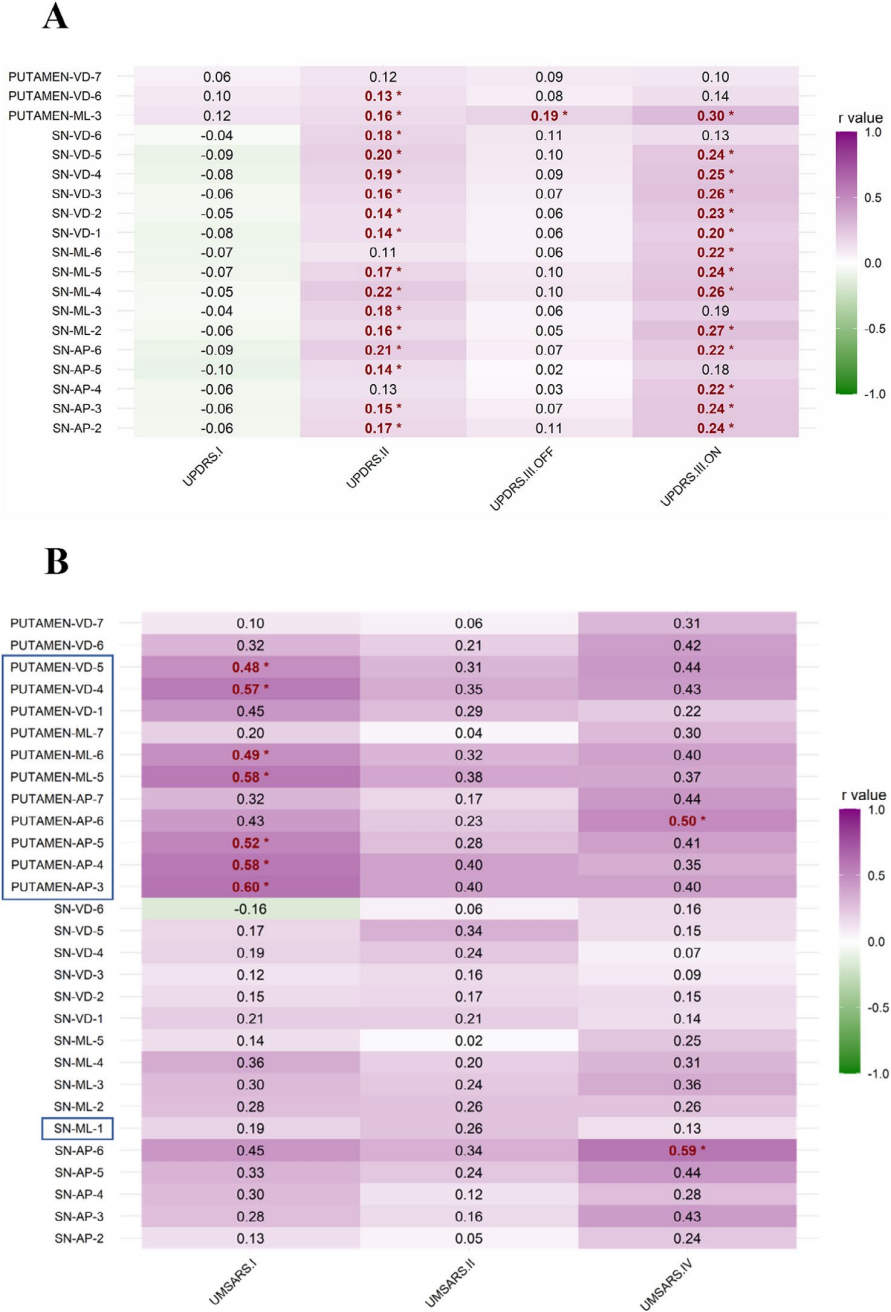
Correlation heatmap of nigrostriatal iron deposition and symptoms of synucleinopathies. Partial correlation analysis of iron content and clinical symptoms in PD (A) and MSA (B), controlling for sex and age. The analysis was performed in the segments with significantly increased iron deposition in PD and MSA compared to OHC. The ROI segments framed in blue (B) were the segments with significantly increased iron deposition in MSA compared to PD. r values that were statistically significant (*p* < 0.05) are bold in red and marked with *. Iron deposition in the SN in PD and putamen in MSA mainly correlated with UPDRS II and UMSARS I scores, respectively. Significant correlations between nigrostriatal iron deposition and on‐state UPDRS part III scores were observed, whereas the correlations in the off‐state UPDRS part III scores were less obvious. A, Anterior; D, Dorsal; L, Lateral; M, Medial; MSA, Multiple system atrophy; OHC, Old healthy controls; *P*, Posterior; PD, Parkinson's disease; QSM, Quantitative susceptibility mapping; SN, Substantia nigra; UMSARS, Unified Multiple System Atrophy Rating Scale; UPDRS, Unified Parkinson's Disease Rating Scale; V, Ventral.

In PD, the QSM values of significantly iron‐loaded segments in the SN and putamen were primarily positively correlated with UPDRS II scores (*p* < 0.05); Significant positive correlations were observed between QSM values of SN and putamen, and on‐state UPDRS part III scores (*p* < 0.05), while only the QSM values of segment 3 of the putamen‐ML axis showed a significant positive correlation with off‐state UPDRS Part III scores (*p* = 0.003) (Figure 4A, detailed statistics can be found in Appendix S6).

In MSA, the QSM values of significantly iron‐loaded segments in the putamen were mainly positively correlated with UMSARS Part I scores (*p* < 0.05); only the QSM values of segment 6 of the SN‐AP axis (*p* = 0.008) and putamen‐AP axis (*p* = 0.029) showed a significant positive correlation with UMSARS Part IV scores. Furthermore, these correlated segments were concentrated in the segments where there was a difference in iron deposition between MSA and PD. (Figure 4B, detailed statistics can be found in Appendix S7).

## 4 Discussion

The present study utilized a combination of QSM and spatial methods to investigate the gradient of neurodegeneration in the nigrostriatal system during healthy aging and in synucleinopathies. The key findings were as follows: (1) similar nigrostriatal iron gradients were consistently observed in the YHC, OHC, PD, and MSA, but with variations in extent; (2) significant iron deposition in all gradient directions was observed in the nigrostriatal system during healthy aging; (3) extensive iron deposition in the SN emerged as a consistent feature in synucleinopathies, with significantly higher iron content in the posterolateral putamen in MSA compared to PD; (4) in PD, iron deposition in the SN predominantly correlated with disease severity (UPDRS part II scores) and on‐state motor symptoms (UPDRS part III scores); in MSA, iron deposition in the putamen was primarily associated with disease severity (UMSARS part I scores).

In YHC, OHC, and synucleinopathies (PD and MSA), the nigrostriatal system iron distribution was not uniform within the same nuclei. Despite this, different populations exhibited similar iron deposition gradients, albeit with varying degrees. To elaborate, an inverted U‐shaped pattern was observed along the SN‐ML, SN‐VD, and caudate‐ML axes. The iron distribution along the SN‐AP, putamen‐ML, and caudate‐AP axes exhibited an S‐shaped or inverted S‐shaped pattern. In the putamen‐AP and putamen‐VD axes, the iron distribution displayed a gradual increase and decrease. These uniform iron distribution patterns within the same nuclei might imply that neurons in different regions of the nucleus differ in the amount of physiological iron required and iron‐related damage in neurodegeneration [32], which were consistent with the heterogeneous deposition of iron found in several previous studies related to subregions of the nucleus [13, 19]. Our study further quantitatively mapped the gradient of iron deposition in the nigrostriatal system during healthy aging and synucleinopathies.

During healthy aging, there was significant and extensive iron deposition within the nigrostriatal system. The increase in brain iron concentration with age may be attributed to various factors, including increased permeability of the blood–brain barrier, inflammation, redistribution of iron within the brain, and changes in iron homeostasis [33, 34]. Previous studies in the elderly population have found that iron is deposited in extensive brain regions such as SN, putamen, caudate nucleus, globus pallidus, and cortex [35, 36, 37]. This was consistent with the extensive iron deposition observed in the nigrostriatal system in the present study. Aging is a major risk factor for neurodegenerative changes. Age‐related iron deposition may be an important factor contributing to neurodegenerative processes [2]. These findings in brain iron content during healthy aging provided a reference framework for investigating pathological iron deposition in age‐related synucleinopathies.

The iron deposition in the SN was more pronounced in both PD and MSA relative to OHC, indicative of nigral iron deposition as a characteristic feature of synucleinopathies, although it is not exclusive to these disorders [38]. Of greater importance, the lateral posterior putamen exhibited a greater degree of iron deposition in MSA compared to PD and OHC, consistent with previous studies and further confirming the established neuropathological differences between MSA and PD [39, 40]. The nigrostriatal system is a common target of damage in PD and MSA. Anatomically, neurons in the SN send long axonal projections to the striatum [41]. Our results suggested that excessive iron deposition in the upstream SN represented a common pathological injury in synucleinopathies, while in MSA, downstream structures like the putamen showed a more severe iron deposition. Excessive iron deposition can induce oxidative stress and activate microglia, promoting α‐synuclein aggregation [42, 43], which plays a crucial role in the neurodegeneration of MSA [43, 44]. Prior susceptibility imaging research has disclosed substantial iron deposition in the putamen of MSA patients, particularly in the posterior region [18, 45, 46, 47, 48], which is similar to our findings and indicates an uneven pathological distribution of iron within this structure. In MSA, the posterolateral portion of the putamen is predominantly affected by neuronal or axonal loss, gliosis, demyelination, and the deposition of ferritin/Fe^3+^ [49]. This study quantitatively confirmed a gradient of iron deposition in the putamen of MSA, that was, iron content gradually increased from the anterior medial to posterior lateral putamen, with higher iron deposition in the posterior lateral region compared to PD and OHC. Whereas, iron deposition of the putamen in PD was not prominent, with only a few segments showing iron deposition. As the disease course of the two synucleinopathies shows no difference, the higher iron content in the posterolateral putamen of MSA compared to PD might indicate more severe damage to neurons in MSA. Clinically, the present study found that excessive iron deposition in the posterolateral putamen of MSA was mainly positively correlated with disease severity (UMSARS part I scores). Excessive iron deposition in the SN in PD was mainly positively correlated with UPDRS part II scores. Interestingly, we observed a significant correlation between nigrostriatal iron deposition and on‐state UPDRS Part III scores, whereas the correlation in the off‐state was less pronounced. This finding suggests that nigrostriatal iron deposition reflected by QSM values could serve as a biomarker for motor symptom progression in the on‐state. Consistent with our results, previous studies have reported a positive correlation between increased nigral iron, reflected by susceptibility or phase values, and on‐state motor scores [50, 51]. However, the relationship between SN iron deposition and both off‐ and on‐state UPDRS Part III scores has shown inconsistency across studies [52, 53, 54], potentially due to variations in iron quantification methods, differences in disease stage among included PD populations, and disease heterogeneity. Notably, the magnitude of correlations between brain iron levels and motor symptoms was far greater for MSA than PD, perhaps suggesting that iron accumulation in PD is more of a “tombstone” phenomenon rather than etiologic. However, this remains debated, requiring longitudinal studies to clarify the causal relationship and mechanistic investigations to uncover its role in disease pathogenesis.

However, current evidence does not support the routine use of deferiprone in PD, as clinical trials have shown iron reduction without symptomatic improvement [55, 56]. Current research has yet to determine whether iron accumulation in the basal ganglia is a causative factor in neurodegeneration or merely a ‘tombstone’ phenomenon. Differences in symptomatic treatment and study design may have influenced these findings—treated patients may mask disease‐modifying effects [57, 58, 59], while untreated patients may worsen due to the absence of therapy [56]. These variations underscore the need for future studies with appropriate population selection, adjustment for concomitant medications, and long‐term follow‐up to better evaluate the therapeutic potential of iron‐modifying therapies in PD and MSA, as well as to clarify whether iron accumulation contributes to neurodegeneration or is a secondary consequence of disease processes.

In contrast to the SN and putamen, iron content in the partial segments of the caudate nucleus was lower in both PD and MSA compared to OHC, and there was no significant difference in iron content in the caudate nucleus between PD and MSA. Previous studies have found that the iron deposition in the caudate nucleus of PD patients tends to be reduced [60]. Currently, our study further uses the gradient method to map the segments of the caudate nucleus with significant iron deposition. The reduction in iron content in the caudate nucleus in synucleinopathies may be attributed to regional iron transport from the caudate nucleus to the putamen, but the underlying pathophysiology remains unclear, requiring further neuropathological studies and external validation to confirm this finding.

This study has several limitations: firstly, there was a small sample size of MSA patients, partially due to its classification as a rare disease. Future research should aim to continue recruiting participants to expand the sample size and validate the current findings. Secondly, this was a cross‐sectional study, and it is necessary to conduct long‐term follow‐ups on existing participants to observe the trajectory of neurodegeneration in various segments of the nigrostriatal system as the disease progresses. Thirdly, the constraints of scanning time result in an anisotropic voxel size for ESWAN images. In future studies, as technology advances, we aim to refine the voxel size to achieve isotropic resolution, which will enhance the precision of QSM quantification.

## 5 Conclusion

This study highlighted the presence of a spatial iron gradient in the nigrostriatal system during healthy aging and synucleinopathies. Iron deposition in all gradient directions occurred in the nigrostriatal system during healthy aging, and this was more evident in the SN in both PD and MSA, with MSA displaying additional iron deposition in the posterolateral putamen. Our findings offer new insights into the heterogeneous iron‐related neurodegeneration within key nuclei, revealing their relationship with manifestations of synucleinopathies.

## Author Contributions

Jiaqi Wen: conception of the research project, organization of the research project, execution of the research project, design of the statistical analysis, execution of the statistical analysis, writing of the first draft manuscript, review and critique of the manuscript. Tao Guo: organization of the research project, design of the statistical analysis, review of the statistical analysis, writing of the first draft manuscript, review and critique of the manuscript. Xiaojie Duanmu: execution of the research project, execution of the statistical analysis, review and critique of the statistical analysis, review and critique of the manuscript. Chenqing Wu, Haoting Wu, Cheng Zhou, Qianshi Zheng, and Weijin Yuan: organization of the research project, execution of the research project, design of the statistical analysis, review and critique of the manuscript. Jianmei Qin, Zihao Zhu, Jingjing Wu, Jingwen Chen, and Jingjing Xu: execution of the research project, review and critique of the statistical analysis, review and critique of the manuscript. Yaping Yan, Jun Tian, Baorong Zhang, Hongjian He, and Minming Zhang: organization of the research project, review and critique of the statistical analysis, review and critique of the manuscript. Xiaojun Guan and Xiaojun Xu: conception of the research project, organization of the research project, design of the statistical analysis, review and critique of the statistical analysis, writing of the first draft manuscript, review and critique of the manuscript.

## Ethics Statement

Our study was approved by the Medical Ethics Committee of the Second Affiliated Hospital of Zhejiang University School of Medicine (ethical approval numbers: 2012–26 and 2017–008).

## Consent

All PD and MSA patients and healthy controls signed informed consent forms in accordance with the approval of the Medical Ethics Committee of the Second Affiliated Hospital of Zhejiang University School of Medicine.

## Conflicts of Interest

The authors declare no conflicts of interest.

## Supporting information


Appendices S1‐S7

## Data Availability

The datasets used and/or analyzed during the current study are available from the corresponding author on reasonable request.

## References

[1] T. J. Collier , N. M. Kanaan , and J. H. Kordower , “Ageing as a Primary Risk Factor for Parkinson's Disease: Evidence From Studies of Non‐Human Primates,” Nature Reviews. Neuroscience 12, no. 6 (2011): 359–366. 21587290 10.1038/nrn3039 PMC3387674

[2] R. J. Ward , F. A. Zucca , J. H. Duyn , R. R. Crichton , and L. Zecca , “The Role of Iron in Brain Ageing and Neurodegenerative Disorders,” Lancet Neurology 13, no. 10 (2014): 1045–1060. 25231526 10.1016/S1474-4422(14)70117-6 PMC5672917

[3] P. I. Semenyuk , “Alpha‐Synuclein Phosphorylation Induces Amyloid Conversion via Enhanced Electrostatic Bridging: Insights From Molecular Modeling of the Full‐Length Protein,” Biophysical Chemistry 307 (2024): 107196. 38335809 10.1016/j.bpc.2024.107196

[4] M. Goedert , R. Jakes , and M. G. Spillantini , “The Synucleinopathies: Twenty Years on,” Journal of Parkinson's Disease 7, no. s1 (2017): S51–s69. 10.3233/JPD-179005 PMC5345650 28282814

[5] A. Hastings , P. Cullinane , S. Wrigley , et al., “Neuropathologic Validation and Diagnostic Accuracy of Presynaptic Dopaminergic Imaging in the Diagnosis of Parkinsonism,” Neurology 102, no. 11 (2024): e209453. 38759132 10.1212/WNL.0000000000209453

[6] A. Fanciulli and G. K. Wenning , “Multiple‐System Atrophy,” New England Journal of Medicine 372, no. 3 (2015): 249–263. 25587949 10.1056/NEJMra1311488

[7] A. Salami , G. Papenberg , R. Sitnikov , E. J. Laukka , J. Persson , and G. Kalpouzos , “Elevated Neuroinflammation Contributes to the Deleterious Impact of Iron Overload on Brain Function in Aging,” NeuroImage 230 (2021): 117792. 33497770 10.1016/j.neuroimage.2021.117792

[8] X. Xu , Q. Wang , and M. Zhang , “Age, Gender, and Hemispheric Differences in Iron Deposition in the Human Brain: An In Vivo MRI Study,” NeuroImage 40, no. 1 (2008): 35–42. 18180169 10.1016/j.neuroimage.2007.11.017

[9] W. Linert and G. N. Jameson , “Redox Reactions of Neurotransmitters Possibly Involved in the Progression of Parkinson's Disease,” Journal of Inorganic Biochemistry 79, no. 1–4 (2000): 319–326. 10830883 10.1016/s0162-0134(99)00238-x

[10] E. Reiter , C. Mueller , B. Pinter , et al., “Dorsolateral Nigral Hyperintensity on 3.0T Susceptibility‐Weighted Imaging in Neurodegenerative Parkinsonism,” Movement Disorders 30, no. 8 (2015): 1068–1076. 25773707 10.1002/mds.26171

[11] J. Zhang , Y. Zhang , J. Wang , et al., “Characterizing Iron Deposition in Parkinson's Disease Using Susceptibility‐Weighted Imaging: An In Vivo MR Study,” Brain Research 1330 (2010): 124–130. 20303339 10.1016/j.brainres.2010.03.036

[12] D. T. Dexter , A. Carayon , F. Javoy‐Agid , et al., “Alterations in the Levels of Iron, Ferritin and Other Trace Metals in Parkinson's Disease and Other Neurodegenerative Diseases Affecting the Basal Ganglia,” Brain 114, no. Pt 4 (1991): 1953–1975. 1832073 10.1093/brain/114.4.1953

[13] X. Guan , Y. Zhang , H. Wei , et al., “Iron‐Related Nigral Degeneration Influences Functional Topology Mediated by Striatal Dysfunction in Parkinson's Disease,” Neurobiology of Aging 75 (2019): 83–97. 30554085 10.1016/j.neurobiolaging.2018.11.013 PMC6538269

[14] G. K. Wenning , I. Stankovic , L. Vignatelli , et al., “The Movement Disorder Society Criteria for the Diagnosis of Multiple System Atrophy,” Movement Disorders 37, no. 6 (2022): 1131–1148. 35445419 10.1002/mds.29005 PMC9321158

[15] A. I. Blazejewska , S. T. Schwarz , A. Pitiot , et al., “Visualization of Nigrosome 1 and Its Loss in PD: Pathoanatomical Correlation and In Vivo 7 T MRI,” Neurology 81, no. 6 (2013): 534–540, 10.1212/WNL.0b013e31829e6fd2. 23843466 PMC3775686

[16] W. Li , B. Wu , and C. Liu , “Quantitative Susceptibility Mapping of Human Brain Reflects Spatial Variation in Tissue Composition,” NeuroImage 55, no. 4 (2011): 1645–1656. 21224002 10.1016/j.neuroimage.2010.11.088 PMC3062654

[17] X. Guan , T. Guo , C. Zhou , et al., “Altered Brain Iron Depositions From Aging to Parkinson's Disease and Alzheimer's Disease: A Quantitative Susceptibility Mapping Study,” NeuroImage 264 (2022): 119683. 36243270 10.1016/j.neuroimage.2022.119683

[18] H. Sjöström , T. Granberg , E. Westman , and P. Svenningsson , “Quantitative Susceptibility Mapping Differentiates Between Parkinsonian Disorders,” Parkinsonism & Related Disorders 44 (2017): 51–57. 28886909 10.1016/j.parkreldis.2017.08.029

[19] M. Azuma , T. Hirai , K. Yamada , et al., “Lateral Asymmetry and Spatial Difference of Iron Deposition in the Substantia Nigra of Patients With Parkinson Disease Measured With Quantitative Susceptibility Mapping,” American Journal of Neuroradiology 37, no. 5 (2016): 782–788. 26822728 10.3174/ajnr.A4645 PMC4867267

[20] N. Pyatigorskaya , C. B. Sanz‐Morère , R. Gaurav , et al., “Iron Imaging as a Diagnostic Tool for Parkinson's Disease: A Systematic Review and Meta‐Analysis,” Frontiers in Neurology 11 (2020): 366. 32547468 10.3389/fneur.2020.00366 PMC7270360

[21] E. Drori , S. Berman , and A. A. Mezer , “Mapping Microstructural Gradients of the Human Striatum in Normal Aging and Parkinson's Disease,” Science Advances 8, no. 28 (2022): eabm1971. 35857492 10.1126/sciadv.abm1971 PMC9286505

[22] A. J. Hughes , S. E. Daniel , L. Kilford , and A. J. Lees , “Accuracy of Clinical Diagnosis of Idiopathic Parkinson's Disease: A Clinico‐Pathological Study of 100 Cases,” Journal of Neurology, Neurosurgery, and Psychiatry 55, no. 3 (1992): 181–184. 1564476 10.1136/jnnp.55.3.181 PMC1014720

[23] R. B. Postuma , D. Berg , M. Stern , et al., “MDS Clinical Diagnostic Criteria for Parkinson's Disease,” Movement Disorders 30, no. 12 (2015): 1591–1601. 26474316 10.1002/mds.26424

[24] W. Li , A. V. Avram , B. Wu , X. Xiao , and C. Liu , “Integrated Laplacian‐Based Phase Unwrapping and Background Phase Removal for Quantitative Susceptibility Mapping,” NMR in Biomedicine 27, no. 2 (2014): 219–227. 24357120 10.1002/nbm.3056 PMC3947438

[25] B. Wu , W. Li , A. Guidon , and C. Liu , “Whole Brain Susceptibility Mapping Using Compressed Sensing,” Magnetic Resonance in Medicine 67, no. 1 (2012): 137–147. 21671269 10.1002/mrm.23000 PMC3249423

[26] H. Wei , R. Dibb , Y. Zhou , et al., “Streaking Artifact Reduction for Quantitative Susceptibility Mapping of Sources With Large Dynamic Range,” NMR in Biomedicine 28, no. 10 (2015): 1294–1303. 26313885 10.1002/nbm.3383 PMC4572914

[27] Y. Guan , J. Xu , H. Wei , X. Xu , and Y. Zhang , “DeepQSMSeg: A Deep Learning‐Based Sub‐Cortical Nucleus Segmentation Tool for Quantitative Susceptibility Mapping,” 45th Annual International Conference of the IEEE Engineering in Medicine and Biology Society 2021 (2021): 3676–3679. 10.1109/EMBC46164.2021.9630449 34892034

[28] D. Georgiev , K. Hamberg , M. Hariz , L. Forsgren , and G. M. Hariz , “Gender Differences in Parkinson's Disease: A Clinical Perspective,” Acta Neurologica Scandinavica 136, no. 6 (2017): 570–584. 28670681 10.1111/ane.12796

[29] I. N. Miller and A. Cronin‐Golomb , “Gender Differences in Parkinson's Disease: Clinical Characteristics and Cognition,” Movement Disorders 25, no. 16 (2010): 2695–2703. 20925068 10.1002/mds.23388 PMC3003756

[30] T. H. Reekes , C. I. Higginson , C. R. Ledbetter , N. Sathivadivel , R. M. Zweig , and E. A. Disbrow , “Sex Specific Cognitive Differences in Parkinson Disease,” NPJ Parkinsons Disease 6 (2020): 7. 10.1038/s41531-020-0109-1 PMC7142103 32284961

[31] E. F. Augustine , A. Pérez , R. Dhall , et al., “Sex Differences in Clinical Features of Early, Treated Parkinson's Disease,” PLoS One 10, no. 7 (2015): e0133002. 26171861 10.1371/journal.pone.0133002 PMC4501841

[32] S. Lee , I. Martinez‐Valbuena , A. E. Lang , and G. G. Kovacs , “Cellular Iron Deposition Patterns Predict Clinical Subtypes of Multiple System Atrophy,” Neurobiology of Disease 197 (2024): 106535. 38761956 10.1016/j.nbd.2024.106535

[33] A. J. Farrall and J. M. Wardlaw , “Blood‐Brain Barrier: Ageing and Microvascular Disease—Systematic Review and Meta‐Analysis,” Neurobiology of Aging 30, no. 3 (2009): 337–352. 17869382 10.1016/j.neurobiolaging.2007.07.015

[34] D. W. Killilea , S. L. Wong , H. S. Cahaya , H. Atamna , and B. N. Ames , “Iron Accumulation During Cellular Senescence,” Annals of the New York Academy of Sciences 1019 (2004): 365–367. 15247045 10.1196/annals.1297.063

[35] L. Zecca , A. Stroppolo , A. Gatti , et al., “The Role of Iron and Copper Molecules in the Neuronal Vulnerability of Locus Coeruleus and Substantia Nigra During Aging,” Proceedings of the National Academy of Sciences of the United States of America 101, no. 26 (2004): 9843–9848. 15210960 10.1073/pnas.0403495101 PMC470762

[36] P. Ramos , A. Santos , N. R. Pinto , R. Mendes , T. Magalhães , and A. Almeida , “Iron Levels in the Human Brain: A Post‐Mortem Study of Anatomical Region Differences and Age‐Related Changes,” Journal of Trace Elements in Medicine and Biology 28, no. 1 (2014): 13–17. 24075790 10.1016/j.jtemb.2013.08.001

[37] B. Hallgren and P. Sourander , “The Effect of Age on the Non‐Haemin Iron in the Human Brain,” Journal of Neurochemistry 3, no. 1 (1958): 41–51. 13611557 10.1111/j.1471-4159.1958.tb12607.x

[38] Y. Zhi , T. Huang , S. Liu , et al., “Correlation Between Iron Deposition and Cognitive Function in Mild to Moderate Alzheimer's Disease Based on Quantitative Susceptibility Mapping,” Frontiers in Aging Neuroscience 16 (2024): 1485530, 10.3389/fnagi.2024.1485530. 39478701 PMC11521800

[39] J. B. Schulz , T. Klockgether , D. Petersen , et al., “Multiple System Atrophy: Natural History, MRI Morphology, and Dopamine Receptor Imaging With 123IBZM‐SPECT,” Journal of Neurology, Neurosurgery, and Psychiatry 57, no. 9 (1994): 1047–1056. 8089667 10.1136/jnnp.57.9.1047 PMC1073125

[40] W. R. Martin , T. E. Roberts , F. Q. Ye , and P. S. Allen , “Increased Basal Ganglia Iron in Striatonigral Degeneration: In Vivo Estimation With Magnetic Resonance,” Canadian Journal of Neurological Sciences 25, no. 1 (1998): 44–47. 10.1017/s0317167100033473 9532280

[41] L. A. Struzyna , K. D. Browne , Z. D. Brodnik , et al., “Tissue Engineered Nigrostriatal Pathway for Treatment of Parkinson's Disease,” Journal of Tissue Engineering and Regenerative Medicine 12, no. 7 (2018): 1702–1716. 29766664 10.1002/term.2698 PMC6416379

[42] M. J. Lee , T. H. Kim , S. J. Kim , et al., “Speculating the Timing of Iron Deposition in the Putamen in Multiple System Atrophy,” Parkinsonism & Related Disorders 63 (2019): 106–110. 30824284 10.1016/j.parkreldis.2019.02.030

[43] J. H. Lee , Y. H. Han , B. M. Kang , C. W. Mun , S. J. Lee , and S. K. Baik , “Quantitative Assessment of Subcortical Atrophy and Iron Content in Progressive Supranuclear Palsy and Parkinsonian Variant of Multiple System Atrophy,” Journal of Neurology 260, no. 8 (2013): 2094–2101. 23670309 10.1007/s00415-013-6951-x

[44] E. Valera and E. Masliah , “The Neuropathology of Multiple System Atrophy and Its Therapeutic Implications,” Autonomic Neuroscience 211 (2018): 1–6. 29169744 10.1016/j.autneu.2017.11.002 PMC5954415

[45] K. Ito , C. Ohtsuka , K. Yoshioka , et al., “Differential Diagnosis of Parkinsonism by a Combined Use of Diffusion Kurtosis Imaging and Quantitative Susceptibility Mapping,” Neuroradiology 59, no. 8 (2017): 759–769. 28689259 10.1007/s00234-017-1870-7

[46] S. Mazzucchi , D. Frosini , M. Costagli , et al., “Quantitative Susceptibility Mapping in Atypical Parkinsonisms,” Neuroimage Clinical 24 (2019): 101999. 31539801 10.1016/j.nicl.2019.101999 PMC6812245

[47] N. Wang , H. Yang , C. Li , G. Fan , and X. Luo , “Using ‘Swallow‐Tail’ Sign and Putaminal Hypointensity as Biomarkers to Distinguish Multiple System Atrophy From Idiopathic Parkinson's Disease: A Susceptibility‐Weighted Imaging Study,” European Radiology 27, no. 8 (2017): 3174–3180. 28105503 10.1007/s00330-017-4743-x

[48] F. J. Meijer , A. van Rumund , B. A. Fasen , et al., “Susceptibility‐Weighted Imaging Improves the Diagnostic Accuracy of 3T Brain MRI in the Work‐Up of Parkinsonism,” American Journal of Neuroradiology 36, no. 3 (2015): 454–460. 25339647 10.3174/ajnr.A4140 PMC8013057

[49] E. Matsusue , S. Fujii , Y. Kanasaki , et al., “Putaminal Lesion in Multiple System Atrophy: Postmortem MR‐Pathological Correlations,” Neuroradiology 50, no. 7 (2008): 559–567. 18463858 10.1007/s00234-008-0381-y

[50] N. He , H. Ling , B. Ding , et al., “Region‐Specific Disturbed Iron Distribution in Early Idiopathic Parkinson's Disease Measured by Quantitative Susceptibility Mapping,” Human Brain Mapping 36, no. 11 (2015): 4407–4420. 26249218 10.1002/hbm.22928 PMC6869507

[51] L. Jin , J. Wang , L. Zhao , et al., “Decreased Serum Ceruloplasmin Levels Characteristically Aggravate Nigral Iron Deposition in Parkinson's Disease,” Brain 134, no. Pt 1 (2011): 50–58, 10.1093/brain/awq319. 21109502

[52] G. E. C. Thomas , L. A. Leyland , A. E. Schrag , A. J. Lees , J. Acosta‐Cabronero , and R. S. Weil , “Brain Iron Deposition Is Linked With Cognitive Severity in Parkinson's Disease,” Journal of Neurology, Neurosurgery, and Psychiatry 91, no. 4 (2020): 418–425. 32079673 10.1136/jnnp-2019-322042 PMC7147185

[53] W. R. Martin , M. Wieler , and M. Gee , “Midbrain Iron Content in Early Parkinson Disease: A Potential Biomarker of Disease Status,” Neurology 70, no. 16 Pt 2 (2008): 1411–1417. 18172063 10.1212/01.wnl.0000286384.31050.b5

[54] M. M. Lewis , G. du , M. Kidacki , et al., “Higher Iron in the Red Nucleus Marks Parkinson's Dyskinesia,” Neurobiology of Aging 34, no. 5 (2013): 1497–1503. 23177595 10.1016/j.neurobiolaging.2012.10.025 PMC3570638

[55] A. Negida , N. M. Hassan , H. Aboeldahab , et al., “Efficacy of the Iron‐Chelating Agent, Deferiprone, in Patients With Parkinson's Disease: A Systematic Review and Meta‐Analysis,” CNS Neuroscience & Therapeutics 30, no. 2 (2024): e14607. 38334258 10.1111/cns.14607 PMC10853946

[56] D. Devos , J. Labreuche , O. Rascol , et al., “Trial of Deferiprone in Parkinson's Disease,” New England Journal of Medicine 387, no. 22 (2022): 2045–2055. 36449420 10.1056/NEJMoa2209254

[57] D. Devos , C. Moreau , J. C. Devedjian , et al., “Targeting Chelatable Iron as a Therapeutic Modality in Parkinson's Disease,” Antioxidants & Redox Signaling 21, no. 2 (2014): 195–210, 10.1089/ars.2013.5593. 24251381 PMC4060813

[58] A. Martin‐Bastida , R. J. Ward , R. Newbould , et al., “Brain Iron Chelation by Deferiprone in a Phase 2 Randomised Double‐Blinded Placebo Controlled Clinical Trial in Parkinson's Disease,” Scientific Reports 7, no. 1 (2017): 1398. 28469157 10.1038/s41598-017-01402-2 PMC5431100

[59] C. Moreau , J. A. Duce , O. Rascol , et al., “Iron as a Therapeutic Target for Parkinson's Disease,” Movement Disorders 33, no. 4 (2018): 568–574. 29380903 10.1002/mds.27275

[60] J. Acosta‐Cabronero , A. Cardenas‐Blanco , M. J. Betts , et al., “The Whole‐Brain Pattern of Magnetic Susceptibility Perturbations in Parkinson's Disease,” Brain 140, no. 1 (2017): 118–131. 27836833 10.1093/brain/aww278

