# Supplementary material for: Gradients of Nigrostriatal Iron Deposition in Healthy Aging and Synucleinopathies
Source: CNS Neurosci Ther. 2025 Mar 25;31(3):e70359. doi: 10.1111/cns.70359 (PMC11933852; doi:10.1111/cns.70359)
Supplement: Supplementary file 1 — Appendices S1‐S7 [file CNS-31-e70359-s001.docx]

**Supplementary materials**

**Supplementary Material 1. Sequence parameters**

Enhanced susceptibility-weighted angiography (ESWAN) images were acquired using gradient recalled echo sequence: repetition time = 33.7 ms; first echo time/spacing/eighth echo time = 4.556 ms/3.648 ms/30.092 ms; flip angle = 20°; field of view = 240 × 240 mm²; matrix = 416 × 384; slice thickness = 2 mm; slice gap = 0 mm; 64 continuous axial slices; iPAT factor = 2; acquisition time = 5 min 9 s.

**Supplementary Material 2. QSM data processing**

The raw phase was unwrapped using a Laplacian-based phase unwrapping [1], and the normalized phase was calculated. The normalized background phase was removed using the spherical-mean-value filtering (V_SHARP) [2]. QSM images were calculated using the STAR-QSM (STreaking Artifact Reduction for QSM) method [3, 4].

**Supplementary material 3. The SVD algorithm for orthogonal axis calculation**

The SVD algorithm solves for

$$M={U \Sigma V}^{*}$$

where M represents an m × 3 matrix of the ROI’s m-centered image coordinates, *Σ* is a diagonal matrix containing the singular values of *M*, and *U* and *V* are matrices with columns representing *M*'s left and right singular vectors, respectively. Consequently, the columns of the 3 × 3 matrix *V* define the principal orthogonal axes of the ROI based on its anatomical shape [5].

In the present study, three orthogonal axes of the SN, putamen, and caudate nucleus were identified as the anterior-posterior (AP), ventral-dorsal (VD), and medial-lateral (ML) axes. The directionality of the SVD-derived axes was determined as follows: the AP axis decreased with the y coordinate, the VD axis increased with the z coordinate, and the ML axis increased with the x coordinate in the right hemisphere and decreased with the x coordinate in the left hemisphere [5].

**Supplementary material 4. Differences in nigrostriatal iron deposition between OHC and YHC.**

| **Variable** | **F** | **P_uncorrected_** | **P_FDR_** |
| --- | --- | --- | --- |
| SN-AP-1 | 109.658 | 0.000 | **0.000** |
| SN-AP-2 | 54.664 | 0.000 | **0.000** |
| SN-AP-3 | 22.831 | 0.000 | **0.000** |
| SN-AP-4 | 7.389 | 0.007 | **0.007** |
| SN-AP-5 | 10.893 | 0.001 | **0.001** |
| SN-AP-6 | 26.706 | 0.000 | **0.000** |
| SN-AP-7 | 48.731 | 0.000 | **0.000** |
| SN-ML-1 | 24.902 | 0.000 | **0.000** |
| SN-ML-2 | 18.804 | 0.000 | **0.000** |
| SN-ML-3 | 13.740 | 0.000 | **0.000** |
| SN-ML-4 | 26.499 | 0.000 | **0.000** |
| SN-ML-5 | 35.508 | 0.000 | **0.000** |
| SN-ML-6 | 51.822 | 0.000 | **0.000** |
| SN-ML-7 | 25.645 | 0.000 | **0.000** |
| SN-VD-1 | 11.299 | 0.001 | **0.001** |
| SN-VD-2 | 3.874 | 0.050 | 0.051 |
| SN-VD-3 | 9.654 | 0.002 | **0.002** |
| SN-VD-4 | 40.831 | 0.000 | **0.000** |
| SN-VD-5 | 93.949 | 0.000 | **0.000** |
| SN-VD-6 | 87.216 | 0.000 | **0.000** |
| SN-VD-7 | 79.108 | 0.000 | **0.000** |
| Putamen-AP-1 | 216.928 | 0.000 | **0.000** |
| Putamen-AP-2 | 200.953 | 0.000 | **0.000** |
| Putamen-AP-3 | 218.240 | 0.000 | **0.000** |
| Putamen-AP-4 | 272.800 | 0.000 | **0.000** |
| Putamen-AP-5 | 284.448 | 0.000 | **0.000** |
| Putamen-AP-6 | 271.921 | 0.000 | **0.000** |
| Putamen-AP-7 | 267.765 | 0.000 | **0.000** |
| Putamen-ML-1 | 118.420 | 0.000 | **0.000** |
| Putamen-ML-2 | 76.384 | 0.000 | **0.000** |
| Putamen-ML-3 | 146.720 | 0.000 | **0.000** |
| Putamen-ML-4 | 292.354 | 0.000 | **0.000** |
| Putamen-ML-5 | 295.908 | 0.000 | **0.000** |
| Putamen-ML-6 | 298.526 | 0.000 | **0.000** |
| Putamen-ML-7 | 316.075 | 0.000 | **0.000** |
| Putamen-VD-1 | 216.692 | 0.000 | **0.000** |
| Putamen-VD-2 | 333.143 | 0.000 | **0.000** |
| Putamen-VD-3 | 323.601 | 0.000 | **0.000** |
| Putamen-VD-4 | 251.706 | 0.000 | **0.000** |
| Putamen-VD-5 | 195.160 | 0.000 | **0.000** |
| Putamen-VD-6 | 135.040 | 0.000 | **0.000** |
| Putamen-VD-7 | 46.305 | 0.000 | **0.000** |
| Caudate-AP-1 | 64.747 | 0.000 | **0.000** |
| Caudate-AP-2 | 62.924 | 0.000 | **0.000** |
| Caudate-AP-3 | 47.430 | 0.000 | **0.000** |
| Caudate-AP-4 | 50.738 | 0.000 | **0.000** |
| Caudate-AP-5 | 108.518 | 0.000 | **0.000** |
| Caudate-AP-6 | 196.097 | 0.000 | **0.000** |
| Caudate-AP-7 | 248.284 | 0.000 | **0.000** |
| Caudate-ML-1 | 1.850 | 0.175 | 0.175 |
| Caudate-ML-2 | 42.370 | 0.000 | **0.000** |
| Caudate-ML-3 | 86.663 | 0.000 | **0.000** |
| Caudate-ML-4 | 116.836 | 0.000 | **0.000** |
| Caudate-ML-5 | 104.608 | 0.000 | **0.000** |
| Caudate-ML-6 | 101.134 | 0.000 | **0.000** |
| Caudate-ML-7 | 79.623 | 0.000 | **0.000** |
| Caudate-VD-1 | 174.151 | 0.000 | **0.000** |
| Caudate-VD-2 | 117.103 | 0.000 | **0.000** |
| Caudate-VD-3 | 38.065 | 0.000 | **0.000** |
| Caudate-VD-4 | 30.855 | 0.000 | **0.000** |
| Caudate-VD-5 | 83.152 | 0.000 | **0.000** |
| Caudate-VD-6 | 102.825 | 0.000 | **0.000** |
| Caudate-VD-7 | 64.928 | 0.000 | **0.000** |

P-values for statistically significant differences after FDR correction are shown in bold. YHC = Young healthy controls; OHC = Old healthy controls; SN = Substantia nigra; A = Anterior; P = Posterior; M = Medial; L = Lateral; V = Ventral; D = Dorsal; FDR = False discovery rate.

**Supplementary material 5. Differences in nigrostriatal iron deposition among OHC, PD, and MSA.**

| **Variable** | **I** | **J** | **MD (I-J)** | **SE** | **P_uncorrected_** | **P_FDR_** | **95% CI for MD** | |
| --- | --- | --- | --- | --- | --- | --- | --- | --- |
| SN-AP-1 | OHC | PD | -0.003 | 0.002 | 0.164 | 0.263 | -0.007 | 0.001 |
|  | OHC | MSA | -0.005 | 0.005 | 0.301 | 0.418 | -0.014 | 0.004 |
|  | MSA | PD | 0.002 | 0.004 | 0.693 | 0.794 | -0.007 | 0.011 |
| SN-AP-2 | OHC | PD | -0.009 | 0.002 | 0.000 | **0.001** | -0.013 | -0.005 |
|  | OHC | MSA | -0.012 | 0.005 | 0.015 | **0.039** | -0.021 | -0.002 |
|  | MSA | PD | 0.003 | 0.005 | 0.567 | 0.691 | -0.007 | 0.012 |
| SN-AP-3 | OHC | PD | -0.010 | 0.002 | 0.000 | **0.001** | -0.015 | -0.005 |
|  | OHC | MSA | -0.016 | 0.005 | 0.003 | **0.010** | -0.026 | -0.005 |
|  | MSA | PD | 0.006 | 0.005 | 0.262 | 0.375 | -0.004 | 0.016 |
| SN-AP-4 | OHC | PD | -0.010 | 0.002 | 0.000 | **0.000** | -0.015 | -0.005 |
|  | OHC | MSA | -0.018 | 0.005 | 0.001 | **0.004** | -0.028 | -0.008 |
|  | MSA | PD | 0.008 | 0.005 | 0.134 | 0.227 | -0.002 | 0.018 |
| SN-AP-5 | OHC | PD | -0.010 | 0.002 | 0.000 | **0.000** | -0.014 | -0.005 |
|  | OHC | MSA | -0.017 | 0.005 | 0.001 | **0.004** | -0.026 | -0.007 |
|  | MSA | PD | 0.007 | 0.005 | 0.144 | 0.239 | -0.002 | 0.016 |
| SN-AP-6 | OHC | PD | -0.012 | 0.002 | 0.000 | **0.000** | -0.016 | -0.008 |
|  | OHC | MSA | -0.018 | 0.004 | 0.000 | **0.001** | -0.026 | -0.009 |
|  | MSA | PD | 0.005 | 0.004 | 0.205 | 0.308 | -0.003 | 0.014 |
| SN-AP-7 | OHC | PD | -0.002 | 0.002 | 0.173 | 0.272 | -0.006 | 0.001 |
|  | OHC | MSA | -0.006 | 0.004 | 0.118 | 0.206 | -0.014 | 0.002 |
|  | MSA | PD | 0.004 | 0.004 | 0.344 | 0.470 | -0.004 | 0.011 |
| SN-ML-1 | OHC | PD | -0.003 | 0.002 | 0.097 | 0.173 | -0.007 | 0.001 |
|  | OHC | MSA | -0.014 | 0.004 | 0.001 | **0.006** | -0.023 | -0.005 |
|  | MSA | PD | 0.011 | 0.004 | 0.014 | **0.037** | 0.002 | 0.019 |
| SN-ML-2 | OHC | PD | -0.010 | 0.002 | 0.000 | **0.001** | -0.015 | -0.005 |
|  | OHC | MSA | -0.022 | 0.005 | 0.000 | **0.001** | -0.032 | -0.011 |
|  | MSA | PD | 0.012 | 0.005 | 0.024 | 0.056 | 0.002 | 0.022 |
| SN-ML-3 | OHC | PD | -0.014 | 0.003 | 0.000 | **0.000** | -0.019 | -0.009 |
|  | OHC | MSA | -0.022 | 0.006 | 0.000 | **0.001** | -0.033 | -0.011 |
|  | MSA | PD | 0.008 | 0.006 | 0.159 | 0.258 | -0.003 | 0.019 |
| SN-ML-4 | OHC | PD | -0.013 | 0.002 | 0.000 | **0.000** | -0.017 | -0.008 |
|  | OHC | MSA | -0.016 | 0.005 | 0.002 | **0.007** | -0.027 | -0.006 |
|  | MSA | PD | 0.004 | 0.005 | 0.448 | 0.561 | -0.006 | 0.014 |
| SN-ML-5 | OHC | PD | -0.009 | 0.002 | 0.000 | **0.000** | -0.014 | -0.005 |
|  | OHC | MSA | -0.013 | 0.005 | 0.004 | **0.013** | -0.022 | -0.004 |
|  | MSA | PD | 0.004 | 0.004 | 0.402 | 0.510 | -0.005 | 0.013 |
| SN-ML-6 | OHC | PD | -0.004 | 0.002 | 0.020 | **0.050** | -0.008 | -0.001 |
|  | OHC | MSA | -0.009 | 0.004 | 0.029 | 0.064 | -0.017 | -0.001 |
|  | MSA | PD | 0.005 | 0.004 | 0.260 | 0.375 | -0.003 | 0.013 |
| SN-ML-7 | OHC | PD | -0.003 | 0.002 | 0.166 | 0.263 | -0.006 | 0.001 |
|  | OHC | MSA | -0.008 | 0.004 | 0.044 | 0.090 | -0.016 | 0.000 |
|  | MSA | PD | 0.006 | 0.004 | 0.165 | 0.263 | -0.002 | 0.013 |
| SN-VD-1 | OHC | PD | -0.008 | 0.002 | 0.001 | **0.004** | -0.012 | -0.003 |
|  | OHC | MSA | -0.016 | 0.005 | 0.001 | **0.004** | -0.026 | -0.007 |
|  | MSA | PD | 0.008 | 0.005 | 0.076 | 0.143 | -0.001 | 0.018 |
| SN-VD-2 | OHC | PD | -0.009 | 0.003 | 0.001 | **0.004** | -0.014 | -0.004 |
|  | OHC | MSA | -0.018 | 0.006 | 0.001 | **0.006** | -0.029 | -0.007 |
|  | MSA | PD | 0.009 | 0.006 | 0.091 | 0.163 | -0.001 | 0.020 |
| SN-VD-3 | OHC | PD | -0.011 | 0.003 | 0.000 | **0.000** | -0.016 | -0.006 |
|  | OHC | MSA | -0.017 | 0.006 | 0.002 | **0.008** | -0.028 | -0.006 |
|  | MSA | PD | 0.006 | 0.005 | 0.246 | 0.364 | -0.004 | 0.017 |
| SN-VD-4 | OHC | PD | -0.010 | 0.002 | 0.000 | **0.000** | -0.014 | -0.005 |
|  | OHC | MSA | -0.014 | 0.005 | 0.003 | **0.011** | -0.023 | -0.005 |
|  | MSA | PD | 0.004 | 0.005 | 0.348 | 0.470 | -0.005 | 0.013 |
| SN-VD-5 | OHC | PD | -0.007 | 0.002 | 0.000 | **0.001** | -0.010 | -0.003 |
|  | OHC | MSA | -0.010 | 0.004 | 0.010 | **0.028** | -0.017 | -0.002 |
|  | MSA | PD | 0.003 | 0.004 | 0.438 | 0.552 | -0.004 | 0.010 |
| SN-VD-6 | OHC | PD | -0.006 | 0.002 | 0.000 | **0.001** | -0.009 | -0.003 |
|  | OHC | MSA | -0.009 | 0.003 | 0.006 | **0.018** | -0.016 | -0.003 |
|  | MSA | PD | 0.003 | 0.003 | 0.352 | 0.472 | -0.003 | 0.010 |
| SN-VD-7 | OHC | PD | -0.003 | 0.001 | 0.031 | 0.068 | -0.006 | 0.000 |
|  | OHC | MSA | -0.006 | 0.003 | 0.043 | 0.089 | -0.012 | 0.000 |
|  | MSA | PD | 0.003 | 0.003 | 0.300 | 0.418 | -0.003 | 0.009 |
| Putamen-AP-1 | OHC | PD | 0.000 | 0.001 | 0.953 | 0.953 | -0.002 | 0.002 |
|  | OHC | MSA | -0.002 | 0.002 | 0.197 | 0.300 | -0.006 | 0.001 |
|  | MSA | PD | 0.003 | 0.002 | 0.181 | 0.278 | -0.001 | 0.006 |
| Putamen-AP-2 | OHC | PD | 0.000 | 0.001 | 0.934 | 0.944 | -0.002 | 0.002 |
|  | OHC | MSA | -0.003 | 0.002 | 0.130 | 0.224 | -0.006 | 0.001 |
|  | MSA | PD | 0.003 | 0.002 | 0.134 | 0.227 | -0.001 | 0.006 |
| Putamen-AP-3 | OHC | PD | -0.001 | 0.001 | 0.257 | 0.373 | -0.003 | 0.001 |
|  | OHC | MSA | -0.006 | 0.002 | 0.004 | **0.012** | -0.011 | -0.002 |
|  | MSA | PD | 0.005 | 0.002 | 0.016 | **0.040** | 0.001 | 0.009 |
| Putamen-AP-4 | OHC | PD | -0.003 | 0.001 | 0.049 | 0.097 | -0.006 | 0.000 |
|  | OHC | MSA | -0.012 | 0.003 | 0.000 | **0.001** | -0.018 | -0.006 |
|  | MSA | PD | 0.009 | 0.003 | 0.002 | **0.008** | 0.003 | 0.015 |
| Putamen-AP-5 | OHC | PD | -0.003 | 0.002 | 0.178 | 0.276 | -0.006 | 0.001 |
|  | OHC | MSA | -0.015 | 0.004 | 0.000 | **0.001** | -0.023 | -0.007 |
|  | MSA | PD | 0.013 | 0.004 | 0.002 | **0.007** | 0.005 | 0.021 |
| Putamen-AP-6 | OHC | PD | 0.003 | 0.002 | 0.252 | 0.370 | -0.002 | 0.007 |
|  | OHC | MSA | -0.018 | 0.005 | 0.001 | **0.004** | -0.028 | -0.007 |
|  | MSA | PD | 0.020 | 0.005 | 0.000 | **0.001** | 0.010 | 0.030 |
| Putamen-AP-7 | OHC | PD | 0.005 | 0.002 | 0.043 | 0.089 | 0.000 | 0.009 |
|  | OHC | MSA | -0.013 | 0.005 | 0.009 | **0.025** | -0.022 | -0.003 |
|  | MSA | PD | 0.017 | 0.005 | 0.000 | **0.002** | 0.008 | 0.027 |
| Putamen-ML-1 | OHC | PD | 0.001 | 0.001 | 0.645 | 0.753 | -0.002 | 0.003 |
|  | OHC | MSA | -0.001 | 0.003 | 0.814 | 0.864 | -0.007 | 0.005 |
|  | MSA | PD | 0.001 | 0.003 | 0.648 | 0.753 | -0.004 | 0.007 |
| Putamen-ML-2 | OHC | PD | -0.002 | 0.001 | 0.055 | 0.108 | -0.004 | 0.000 |
|  | OHC | MSA | -0.001 | 0.002 | 0.773 | 0.854 | -0.005 | 0.003 |
|  | MSA | PD | -0.001 | 0.002 | 0.539 | 0.666 | -0.005 | 0.003 |
| Putamen-ML-3 | OHC | PD | -0.003 | 0.001 | 0.001 | **0.004** | -0.004 | -0.001 |
|  | OHC | MSA | -0.001 | 0.002 | 0.557 | 0.684 | -0.004 | 0.002 |
|  | MSA | PD | -0.002 | 0.002 | 0.309 | 0.426 | -0.005 | 0.002 |
| Putamen-ML-4 | OHC | PD | -0.002 | 0.001 | 0.080 | 0.149 | -0.003 | 0.000 |
|  | OHC | MSA | -0.004 | 0.002 | 0.032 | 0.068 | -0.008 | 0.000 |
|  | MSA | PD | 0.003 | 0.002 | 0.176 | 0.275 | -0.001 | 0.006 |
| Putamen-ML-5 | OHC | PD | -0.001 | 0.001 | 0.368 | 0.478 | -0.004 | 0.001 |
|  | OHC | MSA | -0.009 | 0.003 | 0.001 | **0.006** | -0.015 | -0.004 |
|  | MSA | PD | 0.008 | 0.003 | 0.004 | **0.013** | 0.003 | 0.013 |
| Putamen-ML-6 | OHC | PD | 0.000 | 0.002 | 0.929 | 0.944 | -0.003 | 0.003 |
|  | OHC | MSA | -0.010 | 0.003 | 0.003 | **0.011** | -0.016 | -0.003 |
|  | MSA | PD | 0.010 | 0.003 | 0.003 | **0.009** | 0.003 | 0.016 |
| Putamen-ML-7 | OHC | PD | 0.000 | 0.002 | 0.812 | 0.864 | -0.003 | 0.003 |
|  | OHC | MSA | -0.008 | 0.003 | 0.012 | **0.032** | -0.015 | -0.002 |
|  | MSA | PD | 0.009 | 0.003 | 0.008 | **0.022** | 0.002 | 0.015 |
| Putamen-VD-1 | OHC | PD | 0.000 | 0.002 | 0.932 | 0.944 | -0.004 | 0.004 |
|  | OHC | MSA | -0.014 | 0.004 | 0.002 | **0.008** | -0.022 | -0.005 |
|  | MSA | PD | 0.014 | 0.004 | 0.002 | **0.006** | 0.005 | 0.022 |
| Putamen-VD-2 | OHC | PD | 0.000 | 0.001 | 0.758 | 0.844 | -0.002 | 0.003 |
|  | OHC | MSA | -0.005 | 0.003 | 0.045 | 0.091 | -0.011 | 0.000 |
|  | MSA | PD | 0.006 | 0.003 | 0.030 | 0.065 | 0.001 | 0.011 |
| Putamen-VD-3 | OHC | PD | 0.000 | 0.001 | 0.834 | 0.881 | -0.002 | 0.002 |
|  | OHC | MSA | -0.006 | 0.002 | 0.023 | 0.055 | -0.011 | -0.001 |
|  | MSA | PD | 0.005 | 0.002 | 0.027 | 0.063 | 0.001 | 0.010 |
| Putamen-VD-4 | OHC | PD | -0.001 | 0.001 | 0.359 | 0.478 | -0.003 | 0.001 |
|  | OHC | MSA | -0.007 | 0.003 | 0.005 | **0.015** | -0.012 | -0.002 |
|  | MSA | PD | 0.006 | 0.003 | 0.016 | **0.040** | 0.001 | 0.011 |
| Putamen-VD-5 | OHC | PD | -0.003 | 0.001 | 0.025 | 0.059 | -0.005 | 0.000 |
|  | OHC | MSA | -0.009 | 0.003 | 0.001 | **0.004** | -0.015 | -0.004 |
|  | MSA | PD | 0.007 | 0.003 | 0.014 | **0.037** | 0.001 | 0.012 |
| Putamen-VD-6 | OHC | PD | -0.004 | 0.001 | 0.002 | **0.007** | -0.007 | -0.002 |
|  | OHC | MSA | -0.009 | 0.003 | 0.002 | **0.008** | -0.015 | -0.003 |
|  | MSA | PD | 0.005 | 0.003 | 0.098 | 0.173 | -0.001 | 0.010 |
| Putamen-VD-7 | OHC | PD | -0.008 | 0.002 | 0.000 | **0.000** | -0.012 | -0.005 |
|  | OHC | MSA | -0.013 | 0.004 | 0.001 | **0.004** | -0.020 | -0.005 |
|  | MSA | PD | 0.004 | 0.004 | 0.224 | 0.334 | -0.003 | 0.012 |
| Caudate-AP-1 | OHC | PD | 0.003 | 0.001 | 0.001 | **0.006** | 0.001 | 0.005 |
|  | OHC | MSA | 0.002 | 0.002 | 0.283 | 0.400 | -0.002 | 0.007 |
|  | MSA | PD | 0.001 | 0.002 | 0.678 | 0.781 | -0.003 | 0.005 |
| Caudate-AP-2 | OHC | PD | 0.004 | 0.001 | 0.000 | **0.000** | 0.002 | 0.006 |
|  | OHC | MSA | 0.004 | 0.002 | 0.057 | 0.110 | 0.000 | 0.008 |
|  | MSA | PD | 0.000 | 0.002 | 0.911 | 0.944 | -0.004 | 0.004 |
| Caudate-AP-3 | OHC | PD | 0.003 | 0.001 | 0.000 | **0.001** | 0.002 | 0.005 |
|  | OHC | MSA | 0.003 | 0.002 | 0.088 | 0.159 | 0.000 | 0.007 |
|  | MSA | PD | 0.000 | 0.002 | 0.861 | 0.904 | -0.003 | 0.004 |
| Caudate-AP-4 | OHC | PD | 0.003 | 0.001 | 0.003 | **0.009** | 0.001 | 0.004 |
|  | OHC | MSA | 0.004 | 0.002 | 0.056 | 0.108 | 0.000 | 0.007 |
|  | MSA | PD | -0.001 | 0.002 | 0.606 | 0.724 | -0.005 | 0.003 |
| Caudate-AP-5 | OHC | PD | 0.000 | 0.001 | 0.921 | 0.944 | -0.002 | 0.002 |
|  | OHC | MSA | 0.002 | 0.002 | 0.372 | 0.478 | -0.002 | 0.007 |
|  | MSA | PD | -0.002 | 0.002 | 0.390 | 0.498 | -0.006 | 0.003 |
| Caudate-AP-6 | OHC | PD | -0.003 | 0.001 | 0.029 | 0.064 | -0.006 | 0.000 |
|  | OHC | MSA | -0.005 | 0.003 | 0.124 | 0.215 | -0.010 | 0.001 |
|  | MSA | PD | 0.002 | 0.003 | 0.596 | 0.723 | -0.004 | 0.007 |
| Caudate-AP-7 | OHC | PD | -0.002 | 0.001 | 0.273 | 0.389 | -0.004 | 0.001 |
|  | OHC | MSA | -0.005 | 0.003 | 0.079 | 0.148 | -0.011 | 0.001 |
|  | MSA | PD | 0.004 | 0.003 | 0.205 | 0.308 | -0.002 | 0.010 |
| Caudate-ML-1 | OHC | PD | 0.004 | 0.001 | 0.000 | **0.000** | 0.002 | 0.006 |
|  | OHC | MSA | 0.004 | 0.002 | 0.021 | 0.051 | 0.001 | 0.008 |
|  | MSA | PD | -0.001 | 0.002 | 0.740 | 0.832 | -0.004 | 0.003 |
| Caudate-ML-2 | OHC | PD | 0.005 | 0.001 | 0.000 | **0.000** | 0.003 | 0.006 |
|  | OHC | MSA | 0.005 | 0.002 | 0.007 | **0.020** | 0.002 | 0.009 |
|  | MSA | PD | -0.001 | 0.002 | 0.650 | 0.753 | -0.005 | 0.003 |
| Caudate-ML-3 | OHC | PD | 0.004 | 0.001 | 0.000 | **0.000** | 0.002 | 0.006 |
|  | OHC | MSA | 0.005 | 0.002 | 0.021 | 0.052 | 0.001 | 0.009 |
|  | MSA | PD | 0.000 | 0.002 | 0.804 | 0.863 | -0.004 | 0.003 |
| Caudate-ML-4 | OHC | PD | 0.003 | 0.001 | 0.001 | **0.005** | 0.001 | 0.005 |
|  | OHC | MSA | 0.004 | 0.002 | 0.073 | 0.140 | 0.000 | 0.007 |
|  | MSA | PD | 0.000 | 0.002 | 0.802 | 0.863 | -0.004 | 0.003 |
| Caudate-ML-5 | OHC | PD | 0.000 | 0.001 | 0.636 | 0.751 | -0.001 | 0.002 |
|  | OHC | MSA | 0.001 | 0.002 | 0.602 | 0.724 | -0.003 | 0.004 |
|  | MSA | PD | -0.001 | 0.002 | 0.759 | 0.844 | -0.004 | 0.003 |
| Caudate-ML-6 | OHC | PD | -0.001 | 0.001 | 0.371 | 0.478 | -0.002 | 0.001 |
|  | OHC | MSA | -0.001 | 0.002 | 0.506 | 0.630 | -0.004 | 0.002 |
|  | MSA | PD | 0.000 | 0.002 | 0.800 | 0.863 | -0.003 | 0.004 |
| Caudate-ML-7 | OHC | PD | -0.001 | 0.001 | 0.135 | 0.227 | -0.003 | 0.000 |
|  | OHC | MSA | -0.002 | 0.002 | 0.347 | 0.470 | -0.005 | 0.002 |
|  | MSA | PD | 0.000 | 0.002 | 0.802 | 0.863 | -0.003 | 0.004 |
| Caudate-VD-1 | OHC | PD | 0.007 | 0.001 | 0.000 | **0.000** | 0.005 | 0.010 |
|  | OHC | MSA | 0.007 | 0.003 | 0.011 | **0.030** | 0.002 | 0.012 |
|  | MSA | PD | 0.001 | 0.003 | 0.708 | 0.803 | -0.004 | 0.006 |
| Caudate-VD-2 | OHC | PD | 0.005 | 0.001 | 0.000 | **0.000** | 0.003 | 0.007 |
|  | OHC | MSA | 0.007 | 0.002 | 0.002 | **0.008** | 0.002 | 0.011 |
|  | MSA | PD | -0.002 | 0.002 | 0.365 | 0.478 | -0.006 | 0.002 |
| Caudate-VD-3 | OHC | PD | 0.002 | 0.001 | 0.038 | 0.080 | 0.000 | 0.003 |
|  | OHC | MSA | 0.004 | 0.002 | 0.017 | **0.043** | 0.001 | 0.008 |
|  | MSA | PD | -0.003 | 0.002 | 0.147 | 0.242 | -0.006 | 0.001 |
| Caudate-VD-4 | OHC | PD | 0.000 | 0.001 | 0.636 | 0.751 | -0.001 | 0.002 |
|  | OHC | MSA | 0.001 | 0.002 | 0.709 | 0.803 | -0.003 | 0.004 |
|  | MSA | PD | 0.000 | 0.002 | 0.876 | 0.915 | -0.004 | 0.003 |
| Caudate-VD-5 | OHC | PD | 0.002 | 0.001 | 0.032 | 0.068 | 0.000 | 0.004 |
|  | OHC | MSA | 0.002 | 0.002 | 0.368 | 0.478 | -0.002 | 0.006 |
|  | MSA | PD | 0.000 | 0.002 | 0.921 | 0.944 | -0.004 | 0.004 |
| Caudate-VD-6 | OHC | PD | 0.003 | 0.001 | 0.002 | **0.007** | 0.001 | 0.006 |
|  | OHC | MSA | 0.004 | 0.002 | 0.084 | 0.154 | -0.001 | 0.009 |
|  | MSA | PD | -0.001 | 0.002 | 0.791 | 0.863 | -0.005 | 0.004 |
| Caudate-VD-7 | OHC | PD | 0.006 | 0.001 | 0.000 | **0.000** | 0.003 | 0.008 |
|  | OHC | MSA | 0.006 | 0.003 | 0.028 | 0.063 | 0.001 | 0.011 |
|  | MSA | PD | 0.000 | 0.003 | 0.948 | 0.953 | -0.005 | 0.005 |

P-values for statistically significant differences after FDR correction are shown in bold.

OHC = Old healthy controls; PD = Parkinson’s disease; MSA = Multiple system atrophy. SN = Substantia nigra; A = Anterior; P = Posterior; M = Medial; L = Lateral; V = Ventral; D = Dorsal; FDR = False discovery rate; MD = Mean difference; SE = Standard error; CI = Confidence interval.

**Supplementary material 6. Partial correlation analysis between nigrostriatal iron deposition and symptoms of PD.**

| Covariates: sex & age | UPDRS I | | UPDRS II | | UPDRS III-OFF | | UPDRS III-ON | |
| --- | --- | --- | --- | --- | --- | --- | --- | --- |
|  | r | p | r | p | r | p | r | p |
| SN-AP-2 | -0.061 | 0.360 | 0.167 | **0.011** | 0.106 | 0.110 | 0.241 | **0.015** |
| SN-AP-3 | -0.063 | 0.340 | 0.151 | **0.022** | 0.072 | 0.275 | 0.236 | **0.017** |
| SN-AP-4 | -0.062 | 0.348 | 0.129 | 0.052 | 0.033 | 0.622 | 0.225 | **0.023** |
| SN-AP-5 | -0.095 | 0.153 | 0.145 | **0.029** | 0.025 | 0.705 | 0.185 | 0.062 |
| SN-AP-6 | -0.086 | 0.193 | 0.211 | **0.001** | 0.072 | 0.276 | 0.225 | **0.023** |
| SN-ML-2 | -0.056 | 0.403 | 0.159 | **0.016** | 0.050 | 0.456 | 0.267 | **0.007** |
| SN-ML-3 | -0.042 | 0.530 | 0.185 | **0.005** | 0.055 | 0.405 | 0.194 | 0.051 |
| SN-ML-4 | -0.046 | 0.485 | 0.222 | **0.001** | 0.105 | 0.113 | 0.258 | **0.009** |
| SN-ML-5 | -0.067 | 0.312 | 0.173 | **0.009** | 0.097 | 0.144 | 0.245 | **0.013** |
| SN-ML-6 | -0.073 | 0.269 | 0.112 | 0.091 | 0.060 | 0.364 | 0.225 | **0.023** |
| SN-VD-1 | -0.079 | 0.233 | 0.139 | **0.036** | 0.059 | 0.374 | 0.203 | **0.040** |
| SN-VD-2 | -0.052 | 0.434 | 0.141 | **0.033** | 0.065 | 0.324 | 0.235 | **0.017** |
| SN-VD-3 | -0.057 | 0.395 | 0.165 | **0.013** | 0.074 | 0.264 | 0.261 | **0.008** |
| SN-VD-4 | -0.085 | 0.202 | 0.186 | **0.005** | 0.087 | 0.188 | 0.252 | **0.011** |
| SN-VD-5 | -0.091 | 0.169 | 0.205 | **0.002** | 0.098 | 0.139 | 0.236 | **0.017** |
| SN-VD-6 | -0.038 | 0.571 | 0.183 | **0.006** | 0.108 | 0.102 | 0.134 | 0.181 |
| Putamen-ML-3 | 0.117 | 0.078 | 0.158 | **0.017** | 0.193 | **0.003** | 0.296 | **0.003** |
| Putamen-VD-6 | 0.098 | 0.140 | 0.131 | **0.048** | 0.076 | 0.251 | 0.136 | 0.172 |
| Putamen-VD-7 | 0.063 | 0.342 | 0.116 | 0.079 | 0.088 | 0.186 | 0.102 | 0.310 |

P-values for statistically significant differences are shown in bold. PD = Parkinson’s disease; UPDRS = Unified Parkinson’s Disease Rating Scale; SN = Substantia nigra; A = Anterior; P = Posterior; M = Medial; L = Lateral; V = Ventral; D = Dorsal.

**Supplementary material 7. Partial correlation analysis between nigrostriatal iron deposition and symptoms of MSA.**

| Covariates: sex & age | UMSARS I | | UMSARS II | | UMSARS IV | |
| --- | --- | --- | --- | --- | --- | --- |
|  | r | p | r | p | r | p |
| SN-AP-2 | 0.134 | 0.584 | 0.054 | 0.825 | 0.238 | 0.326 |
| SN-AP-3 | 0.280 | 0.245 | 0.162 | 0.508 | 0.426 | 0.069 |
| SN-AP-4 | 0.297 | 0.217 | 0.124 | 0.612 | 0.279 | 0.248 |
| SN-AP-5 | 0.327 | 0.171 | 0.239 | 0.324 | 0.444 | 0.057 |
| SN-AP-6 | 0.447 | 0.055 | 0.341 | 0.153 | 0.592 | **0.008** |
| SN-ML-1 | 0.187 | 0.442 | 0.256 | 0.289 | 0.132 | 0.590 |
| SN-ML-2 | 0.278 | 0.249 | 0.255 | 0.291 | 0.256 | 0.290 |
| SN-ML-3 | 0.297 | 0.217 | 0.237 | 0.329 | 0.358 | 0.132 |
| SN-ML-4 | 0.358 | 0.133 | 0.202 | 0.407 | 0.313 | 0.191 |
| SN-ML-5 | 0.138 | 0.574 | 0.025 | 0.918 | 0.249 | 0.304 |
| SN-VD-1 | 0.215 | 0.377 | 0.208 | 0.392 | 0.145 | 0.553 |
| SN-VD-2 | 0.151 | 0.536 | 0.172 | 0.482 | 0.151 | 0.538 |
| SN-VD-3 | 0.118 | 0.630 | 0.162 | 0.508 | 0.090 | 0.714 |
| SN-VD-4 | 0.193 | 0.428 | 0.242 | 0.319 | 0.066 | 0.788 |
| SN-VD-5 | 0.174 | 0.476 | 0.335 | 0.161 | 0.150 | 0.541 |
| SN-VD-6 | -0.161 | 0.511 | 0.063 | 0.799 | 0.157 | 0.522 |
| Putamen-AP-3 | 0.600 | **0.007** | 0.399 | 0.090 | 0.399 | 0.090 |
| Putamen-AP-4 | 0.579 | **0.009** | 0.405 | 0.086 | 0.347 | 0.145 |
| Putamen-AP-5 | 0.523 | **0.022** | 0.278 | 0.250 | 0.411 | 0.080 |
| Putamen-AP-6 | 0.435 | 0.062 | 0.228 | 0.348 | 0.500 | **0.029** |
| Putamen-AP-7 | 0.318 | 0.185 | 0.173 | 0.478 | 0.439 | 0.060 |
| Putamen-ML-5 | 0.577 | **0.010** | 0.381 | 0.107 | 0.373 | 0.116 |
| Putamen-ML-6 | 0.486 | **0.035** | 0.317 | 0.187 | 0.399 | 0.090 |
| Putamen-ML-7 | 0.205 | 0.401 | 0.041 | 0.868 | 0.297 | 0.218 |
| Putamen-VD-1 | 0.449 | 0.054 | 0.289 | 0.230 | 0.221 | 0.363 |
| Putamen-VD-4 | 0.567 | **0.011** | 0.352 | 0.139 | 0.427 | 0.068 |
| Putamen-VD-5 | 0.483 | **0.036** | 0.314 | 0.191 | 0.443 | 0.057 |
| Putamen-VD-6 | 0.317 | 0.187 | 0.208 | 0.393 | 0.418 | 0.075 |
| Putamen-VD-7 | 0.100 | 0.684 | 0.062 | 0.801 | 0.311 | 0.195 |

P-values for statistically significant differences are shown in bold. MSA = Multiple system atrophy; UMSARS = Unified Multiple System Atrophy Rating Scale; SN = Substantia nigra; A = Anterior; P = Posterior; M = Medial; L = Lateral; V = Ventral; D = Dorsal.

**References**

1. Li, W., et al., *Integrated Laplacian-based phase unwrapping and background phase removal for quantitative susceptibility mapping.* NMR in biomedicine, 2014. **27**(2): p. 219-27.

2. Wu, B., et al., *Whole brain susceptibility mapping using compressed sensing.* Magnetic resonance in medicine, 2012. **67**(1): p. 137-47.

3. Li, W., B. Wu, and C. Liu, *Quantitative susceptibility mapping of human brain reflects spatial variation in tissue composition.* Neuroimage, 2011. **55**(4): p. 1645-56.

4. Wei, H., et al., *Streaking artifact reduction for quantitative susceptibility mapping of sources with large dynamic range.* NMR Biomed, 2015. **28**(10): p. 1294-303.

5. Drori, E., S. Berman, and A.A. Mezer, *Mapping microstructural gradients of the human striatum in normal aging and Parkinson's disease.* Sci Adv, 2022. **8**(28): p. eabm1971.
